# The Worst Performance Rule, or the Not-Best Performance Rule? Latent-Variable Analyses of Working Memory Capacity, Mind-Wandering Propensity, and Reaction Time

**DOI:** 10.3390/jintelligence8020025

**Published:** 2020-06-02

**Authors:** Matthew S. Welhaf, Bridget A. Smeekens, Matt E. Meier, Paul J. Silvia, Thomas R. Kwapil, Michael J. Kane

**Affiliations:** 1 Department of Psychology, University of North Carolina at Greensboro, Greensboro, NC 27402, USA; mswelhaf@uncg.edu (M.S.W.); basmeek@uncg.edu (B.A.S.); p_silvia@uncg.edu (P.J.S.); 2 Department of Psychology, Western Carolina University, Cullowhee, NC 28723, USA; mmeier@email.wcu.edu; 3 Department of Psychology, University of Illinois at Urbana-Champaign, Champaign, IL 61820, USA; trkwapil@illinois.edu

**Keywords:** the worst performance rule, working memory capacity, mind wandering, lapses of attention, reaction time distributions, ex-Gaussian

## Abstract

The worst performance rule (WPR) is a robust empirical finding reflecting that people’s worst task performance shows numerically stronger correlations with cognitive ability than their average or best performance. However, recent meta-analytic work has proposed this be renamed the “not-best performance” rule because mean and worst performance seem to predict cognitive ability to similar degrees, with both predicting ability better than best performance. We re-analyzed data from a previously published latent-variable study to test for worst vs. not-best performance across a variety of reaction time tasks in relation to two cognitive ability constructs: working memory capacity (WMC) and propensity for task-unrelated thought (TUT). Using two methods of assessing worst performance—ranked-binning and ex-Gaussian-modeling approaches—we found evidence for both the worst and not-best performance rules. WMC followed the not-best performance rule (correlating equivalently with mean and longest response times (RTs)) but TUT propensity followed the worst performance rule (correlating more strongly with longest RTs). Additionally, we created a mini-multiverse following different outlier exclusion rules to test the robustness of our findings; our findings remained stable across the different multiverse iterations. We provisionally conclude that the worst performance rule may only arise in relation to cognitive abilities closely linked to (failures of) sustained attention.

## 1. Introduction

Adults who score higher on intelligence tests also tend to respond faster in simple and choice response time (RT) tasks (Doebler and Scheffler 2016; Jensen 1992; Sheppard and Vernon 2008). However, different parts of the RT distribution are more predictive of cognitive ability: the *worst performance rule* (WPR; Coyle 2003a; Larson and Alderton 1990) describes the empirical finding that subjects’ longest RTs (e.g., the slowest 20% of responses) correlate more strongly with cognitive ability than do their shortest or their average RTs. The WPR appears in a variety of RT tasks (Baumeister and Kellas 1968; Jensen 1982, 1987) and across the lifespan (Coyle 2001, 2003b; Fernandez et al. 2014).

A recent meta-analysis (Schubert 2019) indicated that the WPR is robust: correlations between people’s shortest RTs and intelligence (*r* = −0.18, [95% CI −0.27, −0.08]) were numerically weaker than those between their mean RT and intelligence (*r* = −0.28 [95% CI −0.38, −0.18]) and these were numerically weaker than between their longest RTs and intelligence (*r* = −0.33, [95% CI −0.41, −0.24]). Schubert noted, however, that the meta-analytic results suggested a logarithmic rather than linear association between measures of intelligence and RT. That is, the change between correlations was greatest between shortest and mean RTs, while the change from mean to longest RTs was small. Individual differences in shortest RTs were less strongly associated with ability than were *both* mean and longest RTs. Schubert thus suggested that the WPR be renamed the “not-best performance” rule.

Although the WPR is most often studied in relation to intelligence, related constructs show similar trends. Indeed, the WPR is sometimes explained as reflecting fluctuations of working memory (Larson and Alderton 1990; Larson and Saccuzzo 1989) or of focused attention to the task (Jensen 1992). Failing to maintain attention during a task may result in especially long RTs on those occasional trials where attention is focused elsewhere. People with lower working memory capacity (WMC) and lower intelligence are more prone to attentional lapses (Engle and Kane 2004; Kane and McVay 2012), and WMC appears to be especially related to subjects’ slowest responses (McVay and Kane 2012a; Schmiedek et al. 2007; Unsworth et al. 2010; 2011; 2012; Wiemers and Redick 2019). The attention-control account of the WPR (Larson and Alderton 1990; Unsworth et al. 2010) thus proposes that people of lower ability are more susceptible to attentional lapses that disrupt goal maintenance in working memory than are those of higher ability.

On the one hand, the attention-lapse account of the WPR is consistent with a prominent theory of intelligence, process overlap theory (POT), which proposes that cognitive-task performance requires the contribution of many domain-specific processes and domain-general executive processes (Kovacs and Conway 2016). Central to POT is that, within a cognitive domain, the overlapping processes may compensate for one another, but between domains they cannot; domain-general executive processes may thus act as a bottleneck for item solution when executive demands exceed executive ability. According to POT, then, the WPR arises partly because people with lower WMC/intelligence do not have the ability to meet the necessary executive demands of blocking distractions or sustaining focus on every trial, even though domain-specific processes may be up to the task. These occasions result in extremely slow responses that produce the WPR. On the other hand, POT does not require that the WPR better characterizes performance than does the not-best performance rule. Insofar as other executive processes also contribute to task performance, and these other executive processes tend to fail more frequently than rare attentional lapses (or fail with different thresholds), POT can accommodate either the WPR or the not-best performance rule pattern. Indeed, POT might also predict that ability measures that best capture the propensity for occasional sustained attention failures should show a WPR pattern whereas ability measures that best capture other executive abilities might show a not-best performance rule pattern.

Two approaches have been used most frequently to quantify worst performance. The most common is the ranked-binning procedures, where subjects’ individual RTs are ranked from shortest to longest and split into quantiles (e.g., five bins, from the shortest 20% of RTs to the longest 20%). A second approach models the shape of each subject’s RT distribution. The ex-Gaussian model, for example, represents a subject’s RT distribution—which is typically positively skewed—as a convolution of a Gaussian (normal) and exponential distribution, with three parameters: *mu*, *sigma*, and *tau* (note that the ex-Gaussian model is but one of many that adequately fit RT distributions including the Wald, Gamma, Weibull, and Lognormal functions; Heathcote et al. 2004; Matzke and Wagenmakers 2009; Ulrich and Miller 1993; Van Zandt 2000). In the ex-Gaussian model, mu and sigma reflect the mean and standard deviation of the Gaussian distribution, respectively, whereas tau represents the mean and standard deviation of the exponential component (i.e., the tail of the positively skewed distribution). The parameters of the ex-Gaussian model do not reflect isolated cognitive processes (Matzke and Wagenmakers 2009) but because the tau parameter frequently correlates with normal individual differences in WMC more strongly than do the other parameters, some have proposed that *tau* may sometimes reflect failure of goal maintenance in the form of occasional attentional lapses (McVay and Kane 2012a; Unsworth et al. 2010, 2011, 2012).

If failures of attentional focus can explain the WPR, at least in some task contexts, then assessing subjects’ thought content during a task should also produce patterns consistent with the WPR. During laboratory tasks, as well as in everyday activities, peoples’ thoughts sometimes drift from what they are doing to something unrelated, resulting in the phenomenon of “daydreaming,” “mind wandering,” or “task-unrelated thoughts” (TUTs; e.g., Fox and Christoff 2018; McVay and Kane 2010; Randall et al. 2014; Smallwood and Schooler 2015). TUTs are typically assessed via experience sampling, where subjects are interrupted at unpredictable times during a task or activity and asked to report on their immediately preceding thoughts.

These probed TUT rates have been validated as predicting performance at both within-subject and between-subject levels. At the within-subject level, TUT reports are more frequent following task errors than correct responses (McVay and Kane 2009; Smallwood and Schooler 2006; Stawarczyk et al. 2011) and following relatively fast or variable runs of RTs (Bastian and Sackur 2013; McVay and Kane 2009, 2012a; Seli et al. 2013). TUT reports also vary with assessments of pupil size, an indirect and unobtrusive indicator of arousal and sustained attention (e.g., Unsworth and Robison 2016, 2018; Unsworth et al. 2018), and with particular neuroimaging signatures (e.g., Arnau et al. 2020; Baldwin et al. 2017; Christoff et al. 2009; Kam and Handy 2013). At the between-subjects level, evidence indicates that TUTs reflect, in part, executive abilities to sustain attention. For example, individual differences in probed TUT rate are reliable across tasks and occasions, indicating a trait-like propensity for off-task thought during challenging activities (e.g., Kane et al. 2016; McVay and Kane 2012b; Robison and Unsworth 2018). Moreover, individuals who frequently report TUTs show worse performance (in accuracy, RT variability, or both) on a range of cognitive tasks including reading comprehension (McVay and Kane 2012b; Schooler et al. 2004), working memory (Banks et al. 2016; Kane et al. 2007; Mason et al. 2007; Mrazek et al. 2012; Unsworth and Robison 2015) and attention-control tasks (Cheyne et al. 2009; Kane et al. 2016; McVay and Kane 2009, 2012a, 2012b; Robison et al. 2017). Individual differences in TUT rate and attention-task performance also covary with pupil-size variability in cognitive tasks (e.g., Unsworth and Robison 2017a, 2017b, 2018). These findings, together, indicate that, although it is a self-report measure, TUT rate reflects (at least in part) an ability to sustain attention during challenging tasks.

Several studies have shown that TUT rates correlate with intrasubject variability in RT (i.e., RT standard deviations or coefficients of variation; Bastian and Sackur 2013; McVay and Kane 2009, 2012a; Seli et al. 2013; Unsworth 2015) but only one study has related TUT rates to characteristics of the RT distribution that might be indicative of the WPR. McVay and Kane (2012a) found modest correlations between TUT rates and ranked-bin RTs in a long-duration go/no-go task; subjects with higher TUT rates had shorter RTs in the fastest bins and longer RTs in the slowest bin. From the ranked-bin approach, then, it is unclear whether TUT-variation follows a pure WPR pattern (go/no-go tasks may be unique in eliciting very fast but “mindless” go responses in addition to very slow ones). McVay and Kane also assessed the association between TUT rates and ex-Gaussian parameters, which provided evidence for the WPR: TUT rate was weakly associated with *mu* (*r* = −0.18) and not related to *sigma* (*r* = −0.07), but moderately associated with *tau* (*r* = 0.30); subjects who reported more mind wandering during the task also had more especially long RTs that were captured by the *tau* parameter.

The primary aim of the current study was to apply the meta-analytic findings of Schubert (2019) to a novel dataset, with a relatively large subject sample, across a variety of attention-control tasks, and in relation to two individual-differences constructs—WMC and TUT rate. While the meta-analysis conducted by Schubert (2019) coherently characterized existing “WPR” data, we assessed here whether it would similarly extend to a new, large dataset. Thus, we asked whether there is evidence for the traditional WPR or the “not-best” performance rule pattern (Schubert 2019)—or, perhaps, both, depending on the predictor construct. To do so, we reanalyzed data from a previously published latent-variable study (Kane et al. 2016), focusing on a subset of tasks where RT was a primary dependent measure (using only the non-conflict trials from those response-conflict tasks, in order to make closer contact with the WPR literature). We calculated both ranked-bin and ex-Gaussian parameters and assessed their associations with WMC and TUT rates, both at the individual-task level and at the latent-variable level.

As a secondary aim, we also examined the robustness of our findings to various treatments of outlier trials and outlier subjects via a “mini-multiverse” analysis (Silberzahn et al. 2018; Steegen et al. 2017). One of the main methodological considerations of the WPR, as discussed by Coyle (2003a), is the role of outliers. Given that outliers populate the slowest bins and affect the tau parameter, their inclusion or exclusion might substantially alter measurement of worst performance, and yet Schubert’s (2019) meta-analysis found little consistency in outlier treatment. Here, then, we created different datasets based on different trial-level and subject-level outlier criteria based on commonly reported methods in the studies included in Schubert; we refer to this as a mini-multiverse because we explored a substantial number of reasonable combinations of prototypical outlier treatments without exploring the full universe of all possible treatments and their combinations (which, in terms of RT outlier criteria, are infinite).

## 2. Materials and Methods

### 2.1. Subjects

Kane et al. (2016) enrolled 545 undergraduates into their study from the University of North Carolina at Greensboro, a comprehensive state university (and Minority-Serving Institution for African-American students). Of these, 541 completed the first of three 2 h sessions, 492 completed the second, and 472 completed all three. Full-information maximum-likelihood (ML) estimation was used for missing data (see Kane et al. for details and demographics). By comparison, the average sample size of WPR studies included in Schubert (2019) meta-analysis was 164 (SD = 182), with only one included study testing more than 400 subjects (Dutilh et al. 2017).

### 2.2. Reaction Time (Outcome) Tasks

We focused our analyses on tasks where RT was the primary dependent measure from Kane et al. (2016): The Sustained Attention to Response Task (SART), Number Stroop, Spatial Stroop, Arrow Flanker, Letter Flanker, and Circle Flanker tasks. Below, we briefly describe each task and how their RTs were derived; for analyses reported here, we used only the non-conflict trials from each task.

#### 2.2.1. SART

In this go/no-go task, subjects pressed the space bar for words from one category (*animals*; 89% of trials) but withheld responding to another (*vegetables*; 11% of trials). Subjects completed 675 analyzed trials. RTs were taken from correct responses to “go” (animal) trials.

#### 2.2.2. Number Stroop

Subjects reported the number of digits presented on each trial while ignoring the digits’ identity. Each trial presented 2 to 4 identical digits in a row and subjects responded with one of three labeled keys to indicate the number of digits on screen. There were 300 total trials—of which, 80% were congruent (e.g., *4444*) and the remaining 20% were incongruent (e.g., *2222*). Here, we took RTs from correct responses to congruent trials.

#### 2.2.3. Spatial Stroop

Subjects reported the relative position of a word to an asterisk (left, right, above, below), with the word and asterisk both presented to the left or right, or above or below, fixation. Subjects ignored both the identity of the word (“*LEFT*,” “*RIGHT*,” “*ABOVE*,” “*BELOW”*) and absolute location of the word and asterisk on screen. Subjects responded to the relative position of the word to the asterisk by pressing the corresponding arrow on the numeric keypad arrow keys. Subjects completed a total of 120 trials: 60 presenting words congruent for absolute and relative location, 30 presenting words congruent in absolute location but incongruent with relative location, and 30 presenting words incongruent both in absolute and relative location. Here, RTs were derived from correct responses to trials where words were congruent for both absolute and relative position.

#### 2.2.4. Arrow Flanker

Subjects reported the direction of a centrally presented arrow (“<” vs. “>”) via keypress, with the arrow flanked horizontally by 4 distractors. Subjects completed two blocks of 96 trials: 24 neutral trials (target arrow presented amid dots), 24 congruent trials (all arrows pointing the same direction), 24 stimulus-response incongruent trials (central arrow pointing opposite direction of flankers), and 24 stimulus-stimulus incongruent trials (central arrow presented amid upward pointing arrows). Here, we used RTs from correct responses to both neutral and congruent trials.

#### 2.2.5. Letter Flanker

Subjects reported whether a centrally presented “F” appeared normally or backwards via keypress, with that Letter Flanker horizontally by 6 distractors. Subjects completed 144 trials: 24 neutral trials (normal or backwards F presented amid dots), 48 congruent trials (target and distractor Fs all facing the same direction), 24 stimulus-response incongruent trials (target facing opposite direction of distractors), and 24 stimulus-stimulus incongruent trials (target presented amid right- and left- facing Es and Ts tilted at 90 and 270 degrees). Here, RTs were derived from correct responses to neutral and congruent trials.

#### 2.2.6. Circle Flanker

Subjects reported whether a target letter was an X or N, via keypress, with the target flanked by two distractors. Targets appeared in one of eight possible locations in a circle, with distractors appearing to position one either side of the target; all other location were occupied by colons. Subjects completed 160 trials: 80 neutral trials (target letter surrounded by colons) and 80 stimulus-stimulus conflict trials (target flanked by two different distractors from the set H, K, M, V, Y, Z). Here, we took RTs from correct responses to neutral trials.

### 2.3. Cognitive Predictor Measures

For a detailed description of the tasks used for the present analyses (as well as non-analyzed tasks and task order), see Kane et al. (2016). Here, we used only two of their cognitive constructs as predictors in our statistical models—WMC and TUT rate (i.e., we did not analyze performance from attention-constraint or attention-restraint tasks here, other than the neutral and congruent RTs described from the tasks above as outcome measures).

#### 2.3.1. Working Memory Capacity (WMC)

In six tasks, subjects briefly maintained items in memory while engaging in secondary tasks or mental updating. Four complex span tasks presented sequences of verbal or visuospatial items that required immediate serial recall (Operation Span, Reading Span, Symmetry Span, Rotation Span). Memory items were preceded by unrelated processing tasks requiring yes/no responses. Two memory-updating tasks (Running Span, Updating Counters) required subjects to maintain an evolving set of stimuli in serial order while disregarding previous stimuli. Higher scores indicated more accurate recall.

#### 2.3.2. Thought Reports of TUT

Thought probes appeared randomly within 5 tasks (45 in SART, 20 in Number Stroop, 20 in Arrow Flanker, 12 in Letter Flanker, and 12 in an otherwise-unanalyzed 2-back task). At each probe, subjects chose among eight presented options that most closely matched the content of their immediately preceding thoughts. TUTs were comprised of response options 3–8 in Kane et al. (2016): “*Everyday Things*” (thoughts about normal life concerns, goals, and activities); “*Current State of Being*” (thoughts about one’s physical, cognitive, or emotional states); “*Personal Worries*” (thoughts about current worries); “*Daydreams*” (fantastical, unrealistic thoughts); “*External Environment*” (thoughts about things or events in the immediate environment); “*Other*.”

### 2.4. RT Data Cleaning Procedure

All data were cleaned and aggregated in R (R Core Team 2018) using the *dplyr* package (Wickham et al. 2018). Data from all RT tasks were cleaned in the same manner for primary analyses. We first identified and removed error and post-error trials (and, in tasks that included thought probes, post-probe trials). In tasks that included conflict trials, we removed all conflict trials to focus our analyses on non-conflict trials to remove potential interference effects. From the remaining trials, we eliminated likely anticipatory trials (i.e., faster than 200 ms). For all primary regression and latent-variable models, we next identified trial outliers that were outside 3 times the interquartile range (3*IQR) of each individual subjects’ mean RT for each task and replaced those trials with values equal to 3*IQR. This procedure affected <2% of trials in each task. Following all trial-level treatments and aggregation, RT variables were z-scored at the sample level. As we will discuss later, a mini-multiverse analyses repeated our primary latent-variable analyses across various combinations of trial- and subject-level outlier decisions (see Section 3.3.1).

## 3. Results

Data used for all analyses, as well as analysis scripts and output, are available via the Open Science Framework (https://osf.io/9qcmx/). For detailed description of data-analysis exclusions, scoring of predictor tasks, and treatment of outliers in predictor tasks, please see Kane et al. (2016). We modeled the cognitive predictor constructs (WMC and TUTs) identically to Kane et al., including any residual correlations among indicators.

In the following sections, we first report results from the ranked-bin approach. Regression analyses provide descriptive evidence of the WPR in each task separately. Our main results assess latent-variable models for RT ranked bins and their correlations with WMC and TUTs. We follow these results with latent-variable models using ex-Gaussian parameters to assess the WPR (via the tau parameter). Lastly, we present a mini-multiverse analysis to explore whether varying treatments of outliers influence the robustness of our primary latent-variable analyses.

### 3.1. Ranked-Bin Analyses

#### 3.1.1. Descriptive Statistics and Zero-Order Correlations

Table 1 presents descriptive statistics for all ranked-bin measures. Mean RTs increased substantially across bins for all tasks, and standard deviations suggest considerable between-subject variation (also increasing over bins). Supplemental Table S1 presents zero-order correlations among the predictor and RT-outcome measures. Correlations among RTs from the same bins across different tasks (e.g., SART Bin 5, Arrow Flanker Bin 5) were modest, suggesting convergent validity among ranked-bin RTs. It thus appears that we measured a reasonably trait-like pattern in RT distributions across subjects.

#### 3.1.2. Regression Evidence for the Worst Performance Rule

We first present two sets of regression analyses to assess descriptive evidence for either the WPR or the not-best performance rule (Schubert 2019) across the RT tasks. The first set of regressions tested whether WMC, TUT rates, or both, interacted with RT quantile bin to predict RT. The WPR would be reflected in associations with WMC and/or TUTs getting stronger across the bins. That is, WMC- and TUT-related differences should be largest in subjects’ slowest RT bin (i.e., Bin 5). Alternatively, evidence for the not-best performance rule would come in the form of associations with WMC and/or TUTs increasing across subjects’ fastest and “mean” RT bins (i.e., Bin 1 and Bin 2), but the slopes from “mean” to slowest RT bins should look similar. As seen in Table 2 (under the Model 1 column), across tasks, Bin was a significant predictor of RT (as it should have been, by design); RTs were longer at the later than earlier bins. WMC was also a significant predictor of RT in all tasks, except the SART. However, all tasks exhibited a significant Bin × WMC interaction. Supplemental Figure S1 depicts this interaction for each task. The relation between WMC and RT in the SART was unique, in that extremely short RTs, which likely reflect habitual “go” responding, were positively related to WMC. That is, higher-WMC subjects’ shortest RTs were longer than lower-WMC subjects’ shortest RTs, consistent with prior research (McVay and Kane 2009). As can be seen in Supplemental Figure S1, across many of the tasks, the beta coefficients numerically increased across the bins. However, across the tasks, the 95% confidence intervals tended to overlap across many of the non-fastest bins (i.e., 2 through 5). This suggests that subjects’ mean to longest RTs might not be statistically different in their association to WMC, perhaps inconsistent with the WPR. In interpreting these patterns, however, it is important to note that when RTs are highly correlated across bins (see Supplemental Table S1 for correlations) and variability increases across bins, the regression slopes must also increase across bins (Frischkorn et al. 2016). Thus, the slope increases we see across bins might be artifacts and not sufficient evidence for the WPR.

We next ran the same analyses using TUT rates as our ability predictor. As seen in Table 2 (under the Model 2 column), Bin again predicted RT across the tasks, as it must. TUT rates significantly predicted RT in all the tasks except for SART and Spatial Stroop. Of most importance, the TUT × Bin interaction was significant across the tasks (Supplemental Figure S2 visualizes the interaction for each task). Again, we find a unique pattern of results in the SART: higher TUT rates were associated with shorter RTs in subjects’ fastest bins (e.g., Bin 1 and 2), likely reflecting absentminded “go” responding. Consistent across the tasks, though, we found that higher TUT rates associated with longer RTs in subjects’ slowest bins (e.g., Bins 3–5). In many of the tasks, Bin 5 and Bin 4 had overlapping confidence intervals. However, the Bin 5 confidence intervals often failed to overlap with Bin 3, suggesting that the association between TUT rate and RT was strongest for the longest RTs versus the mean RTs. Thus, when using TUT rate as our measure of ability, we find stronger descriptive evidence for the WPR than we did for WMC.

In the next set of regression analyses, we investigated the predictive power of RT bins on WMC and TUTs. Hierarchical linear regressions tested whether RT bins for the slowest quintiles predicted variation in WMC and TUTs after accounting for the fastest RT quintiles. Given the strong correlations between adjacent bins in each task (e.g., Bin 1 and Bin 2), we focused these and all subsequent analyses on Bin 1, Bin 3, and Bin 5. This approach also parallels Schubert’s (2019) focus on “fast RT” (i.e., Bin 1), “mean RT” (i.e., Bin 3), and “slow RT” (i.e., Bin 5).

If the longest RTs are the ones that are especially related to WMC and TUT (i.e., typical WPR findings), then the slowest RT bins should account for unique variance in WMC and TUT rate after accounting for subjects’ fastest and mean RT bins. Table 3 shows the results of hierarchical regressions on WMC, which suggest that the slower bins do not add much predictive power beyond the faster bins. That is, after adding in Bins 3 and 5 to the models, Bin 1 or Bin 3 (or both) were the main predictors of WMC, rather than Bin 5. (We note the evidence of suppressor effects in many of the final models of each task; Bin 1 negatively predicted WMC in the initial models for each task, but that effect sometimes changed sign once the slower bins are added into the models.) Overall, then, when WMC serves as the outcome, it appears that we have better evidence for the not-best performance rule (Schubert 2019) than for the WPR. 

Table 4 shows the parallel regression analyses for the TUT rate outcome. Here, TUTs were solely predicted by the slowest RT bins in several of the tasks. These TUT-related finding are more in line with the WPR than with the not-best performance rule. At the task level, then, it appears that we find evidence suggestive of either the WPR or the not-best performance rule, depending on the cognitive ability being assessed (not-best performance for WMC associations, worst performance for TUT rate associations).

#### 3.1.3. Confirmatory Factor Analyses of Ranked Bins

We next assessed how binned RTs correlated with our cognitive predictors at the latent-variable level. Like the above regression models, we included only RT Bins 1, 3, and 5 to best parallel Schubert’s (2019) meta-analytic findings (and to circumvent problems from extremely strong correlations between adjacent RT bins). A measurement model for just RT Bins 1, 3, and 5 fit the data well, *χ*^2^ /df = 2.40, CFI = 0.977, TLI = 0.970, RMSEA = 0.051 [0.043–0.059], SRMR = 0.052, indicating consistent individual differences in RT bins across our tasks. Even after dropping adjacent bins, however, some of the bins were highly correlated with each other, especially the closer bins (φ_bin1,3_ = 0.94; φ_bin3,5_ = 0.92). The correlation between Bin 1 and Bin 5 (φ_bin1,5_ = 0.76) was still strong, but was numerically weaker than those of the closer bins.

**Figure 1:**
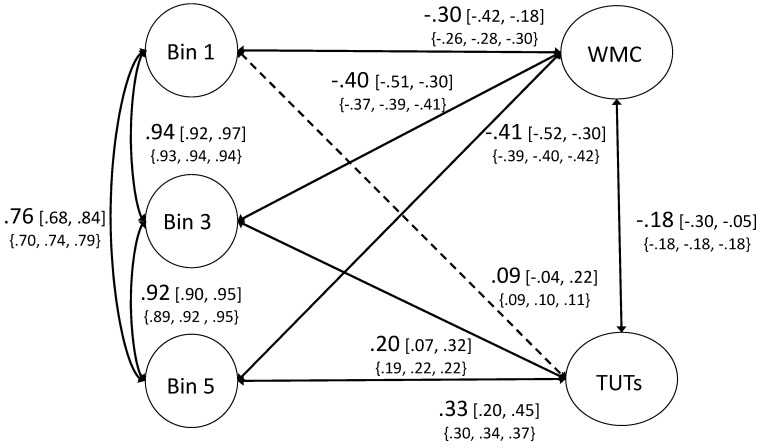
Confirmatory factor analysis of ranked-bin models. WMC = working memory capacity. TUTs = task-unrelated thoughts. Path estimates are presented in largest size font. The 95% confidence intervals are presented in brackets. Values in the braces below represent the lowest, median, and highest estimate from the mini multiverse analysis (see Section 3.3). For clarity, factor loadings are not presented here; see Supplemental Table S3 for factor loadings for all models included in the primary analyses.

Next, we asked how these factors correlated with WMC and TUT rates. Prior work on the WPR would suggest that cognitive abilities should correlate more strongly with the slowest RT bins than with the rest of the RT distribution. However, Schubert’s (2019) meta-analysis suggested that an individual’s cognitive ability is equally correlated with their mean RT and longest RTs, with both correlations stronger than with subjects’ shortest RTs. A confirmatory factor analysis with WMC, TUTs, and RT bins (1, 3, 5) fit the data well, *χ*^2^/df = 2.03, CFI = 0.964, TLI = 0.957, RMSEA = 0.044 [0.039–0.048], SRMR = 0.062. Figure 1 presents the full model. WMC was significantly negatively correlated with each RT bin. Of most importance, WMC appeared to be less strongly correlated with Bin 1 (φ = −0.30), than with Bin 3 or Bin 5 (φs = −0.40 and −0.41, respectively). To test whether these estimates were statistically different from each other, we ran another CFA where the paths from WMC to Bin 1 and Bin 3 were set to be equal. Although this model fit the data well, *χ*^2^/df = 2.24, CFI = 0.962, TLI = 0.956, RMSEA = 0.048 [0.044–0.053], SRMR = 0.065, it fit significantly worse than the model with all paths freely estimated, *χ*^2^_diff_ = 19.99, df_diff_ = 1, *p* < 0.001. WMC correlated less strongly with Bin 1 RTs than with the others, thus demonstrating the not-best performance rule.

For TUT-rate correlations, in contrast, we find a pattern more consistent with the WPR. TUTs were not significantly related to subjects’ fastest RT bin (φ = 0.09, *p* > 0.05), but they were to subjects’ middle RT bin (φ = 0.20, *p* < 0.05) and slowest RT bin (φ = 0.33, *p* < 0.01). Here, we tested whether fixing the paths from TUTs to Bin 3 and Bin 5 to be equal significantly hurt model fit. In fact, fixing these correlations to be equal significantly hurt model fit, *χ*^2^_diff_ = 8.49, df_diff_ = 1, *p* < 0.005. Therefore, the pattern of correlations does appear to get stronger across the RT bins, consistent with traditional WPR findings. These results complement the task-based regression analyses and suggest that evidence for the WPR and not-best performance rule depend on the cognitive ability construct being measured. Those abilities that are most closely tied to attentional lapses (i.e., TUTs) show more consistent evidence for the WPR, whereas those less strongly related to lapses (i.e., WMC) tend to show the not-best performance pattern. (As a secondary approach, we attempted to fit latent growth curve models to the ranked bin data (Duncan et al. 2006; Preacher et al. 2008), but we were unable to fit the data with these models, likely as a result of the high collinearity between the bin factors.)

### 3.2. Ex-Gaussian Analyses

#### 3.2.1. Descriptive Statistics and Zero-Order Correlations

As a second methodological approach to characterizing RTs (and worst performance), we used ex-Gaussian models to estimate three parameters from subjects’ RT distributions for each of the tasks, *mu*, *sigma*, and *tau*. We conducted ex-Gaussian modeling with the *retimes* package (Massidda 2013). Table 5 provides the descriptive statistics for the ex-Gaussian parameter estimates for each task. Supplemental Table S2 shows the bivariate correlations among the cognitive predictors and ex-Gaussian parameter estimates. Each parameter appeared to be modestly correlated across tasks, suggesting convergent validity, and in most cases each parameter correlated more strongly with its counterparts across tasks than with the other parameters across tasks, suggesting discriminant validity. Thus, as with RT bins, it appears that we measured trait-like patterns in ex-Gaussian RT. 

#### 3.2.2. Ex-Gaussian Structural Models

**Figure 2:**
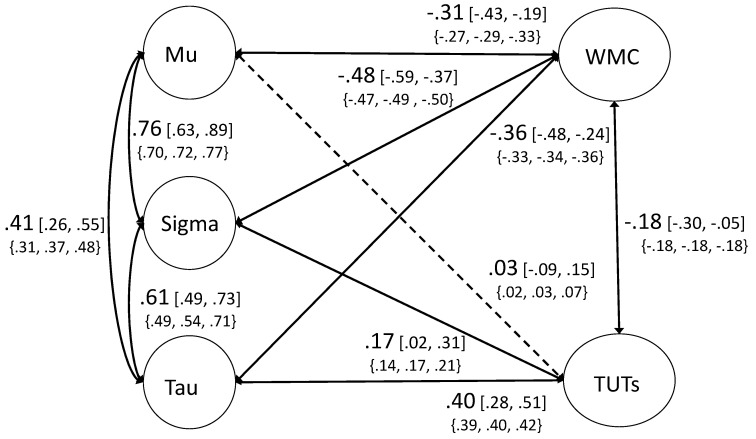
Confirmatory factor analysis of ex-Gaussian model. WMC = working memory capacity. TUTs = task-unrelated thoughts. Path estimates are presented in largest size font. The 95% confidence intervals are presented in brackets. Values in the braces below represent the lowest, median, and highest estimate from the mini multiverse analysis (see Section 3.3). For clarity, factor loadings are not presented here; see Supplemental Table S3 for factor loadings for all models included in the primary analyses.

We next attempted to model latent variables from the ex-Gaussian variables. Model fit was acceptable, *χ*^2^ /df = 2.74, CFI = 0.940, TLI = 0.920, RMSEA = 0.052 [0.044–0.059], SRMR = 0.066. Positive correlations among the ex-Gaussian factors were moderate to strong, in line with prior work using this technique (e.g., Schmiedek et al. 2007). We next added both WMC and TUTs into the model as a confirmatory factor analysis. This model fit the data adequately, *χ*^2^/df = 2.15, CFI = 0.920, TLI = 0.905, RMSEA = 0.046 [0.042–0.051], SRMR = 0.065. As seen in Figure 2, WMC correlated significantly and negatively with each parameter estimate, not just with tau. These estimates do not follow a worst-performance rule pattern (i.e., the correlation with mu is substantial, and the strongest WMC correlation is with sigma rather than tau). We tested whether fixing the paths between WMC and mu and tau significantly hurt model fit; it did not, *χ*^2^_diff_ = 0.12, df_diff_ = 1, *p* > 0.05. TUT rates showed a different pattern. TUT rate was not significantly correlated with mu (φ = 0.03) and was weakly associated with sigma (φ = 0.17). Importantly, however, TUT rate was moderately correlated with tau (φ = 0.40). As we did with WMC, we tested whether fixing the paths between TUTs and mu and tau hurt model fit, and here it did, *χ*^2^_diff_ = 27.64, df_diff_ = 1, *p* < 0.001. This suggests that subjects who were more prone to lapses of attention associated with mind wandering also had more behavioral lapses (i.e., especially long RTs) captured by the tau parameter. Thus, it again appears that TUT-rate variation shows the worst-performance rule pattern.

### 3.3. Mini-Multiverse Analysis of WPR Findings

Researchers that conduct binning and ex-Gaussian analyses of RTs have many degrees of freedom in how they treat the data corresponding to the upper limit of the RT distribution. While some relatively long RTs may be characteristic of an attentional lapse, it is possible that other, perhaps outlying, RTs result from idiosyncratic or unplanned events (e.g., sneezes, looking away from the monitor, checking a phone) that aren’t characteristic of a subject’s performance or ability. How should the data analyst handle these long or outlying RTs, particularly when WPR-related phenomena are driven by exactly those longer-than-average RTs? There is no single answer. While many WPR studies report some RT outlier treatment, there are almost as many treatment variations as there are studies. In just the 23 studies included in Schubert’s (2019) meta-analysis, nine papers did not describe any RT outlier treatment and the remaining 14 each had different criteria and protocols. Some of these treatments were simple (e.g., removing the slowest RT trial), while others were more complex (e.g., an iterative process that removed outlying trials until none remained). The most common approach was that of defining a cutoff based on each subjects’ own RT distribution (e.g., mean RT + 3.5*SD) and discarding trials that were slower than this criterion.

**Figure 3:**
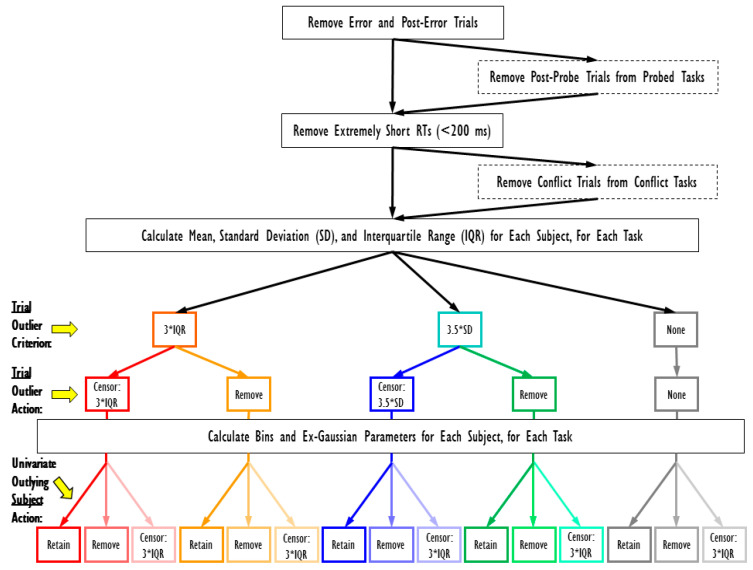
Mini-multiverse decision tree. Solid black boxes represent decisions that were made in every task in every multiverse iteration. Dashed black boxes include decisions that were made in some tasks (e.g., those with thought probes or conflict trials) in every multiverse iteration. Retain = kept outlier in dataset. Remove = remove outlier (trial or subject) from dataset. Censor = change outlying value to specified cutoff.

Differences in cutoff values for outlying RTs might alter RT distributions, and their correlations with cognitive abilities, across studies. To examine this possibility, we created a mini-multiverse of potential datasets based on various outlier cutoff criteria and consequence (see Steegen et al. 2017); we describe this as a mini-multiverse because we did not assess every possible combination of possible (or plausible) data treatments. The processing of data is an active process in which many decisions can be made (e.g., outlier cutoffs). Thus, the raw dataset that researchers begin with can ultimately yield different datasets based on different outlier decisions (i.e., multiverses). To increase transparency and test the robustness of our main latent-variable findings, we created variations of the original dataset based on different RT cutoff values for outliers (e.g., mean RT + 3*IQR; mean RT + 3.5*SD) and whether trials outside of those cutoffs were either (a) removed completely or (b) censored to the cutoff value before aggregating. We also created versions that took into account the potential impact of univariate outlier subjects after aggregating the data. This univariate outlier rule was based on 3*IQR and was used across all multiverse paths. Figure 3 depicts our decisions in creating the multiverse. Again, these decisions are not exhaustive, and an infinite set of other cutoffs could be plausibly chosen (e.g., mean RT + 2.5*SD, mean RT + 2.75*SD, and mean RT + 3*SD). To foreshadow, our findings were impressively consistent across different iterations of the multiverse, suggesting that deviations across our decisions did not affect our outcomes and conclusions. Whether this is generally true, at least in studies with large sample sizes that take a latent-variable approach across multiple RT indicators, remains to be determined by multiverse analyses of other studies.

#### 3.3.1. Mini-Multiverse Results

**Figure 4:**
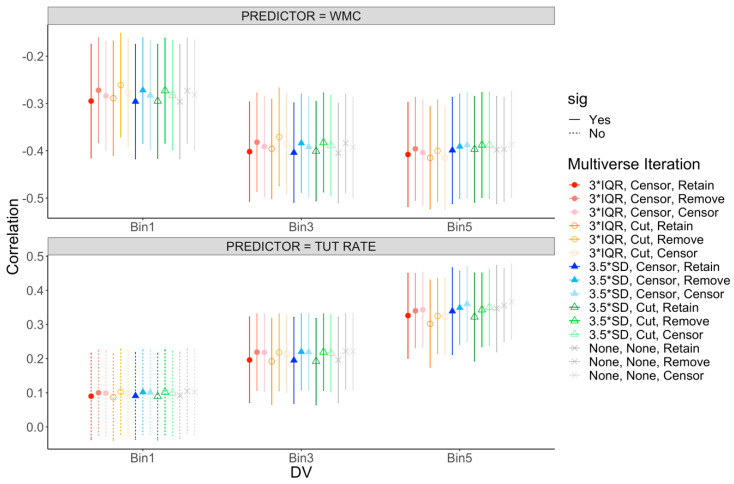
Mini-multiverse of ranked-bin correlations. The top panel presents correlations with working memory capacity (WMC). The bottom panel presents correlations with rate of task-unrelated thought (TUT). Points reflect the correlation, with error bars representing the 95% confidence interval (CI) around the estimate. Circles represent iterations where outlying trials were defined by interquartile ranges (IQR), triangles represent iterations where outlying trials were defined by standard deviations (SDs), and xs represent iterations where no criteria were applied to outlying trials. Filled shapes reflect iterations where outlying trials were censored to the respective cutoff value before aggregating and open shapes reflect iterations where outlying trials were removed before aggregating. Colors presented in this figure match those illustrating the multiverse iterations in Figure 3. Solid CIs represent significant correlations, and dashed CIs represent non-significant correlations at *p* = 0.05.

Supplemental Table S4 presents the latent correlations among WMC, TUT rates, and our Bin factors across the various multiverse iterations. These results are visually depicted in Figure 4. Estimates of these associations are remarkably stable across iterations, with correlations within a range of +/− 0.06. Thus, changing the outlier cutoff for individual trials, cutting, censoring, or retaining those outlier trials, and deciding whether or not univariate outliers should be included, cut, or censored did not substantively alter the estimates of the relations between our cognitive ability factors and RT bins. As in our main analysis reported above, WMC was negatively related to each RT Bin, and this pattern reflected the not-best performance rule—WMC showed weaker correlations with subjects’ shortest RTs and numerically similar estimates for subjects’ mean and longest RTs. As well, the association between TUT rate and the RT bins followed an identical pattern to the main analyses: TUT rates were not related to subjects’ shortest RTs, were weakly associated with subjects’ mean RTs, but were more strongly related to subjects’ longest RTs. Thus, across the mini-multiverse, we see evidence for the WPR only when examining TUT propensity as our cognitive ability measure.

**Figure 5:**
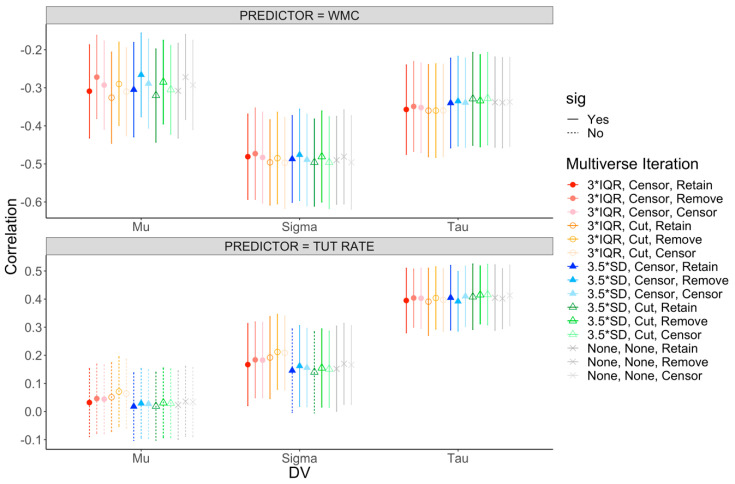
Mini-multiverse of ex-Gaussian correlations. The top panel presents correlations with working memory capacity (WMC). The bottom panel presents correlations with rate of task-unrelated thoughts (TUTs). Points reflect the correlation, with error bars representing the 95% confidence interval (CI) around the estimate. Circles represent iterations where outlying trials were defined by interquartile ranges (IQR), triangles represent iterations where outlying trials were defined by standard deviations (SDs), and xs represent iterations where no criteria were applied to outlying trials. Filled shapes reflect iterations where outlying trials were censored to the respective cutoff value before aggregating and open shapes reflect iterations where outlying trials were removed before aggregating. Colors presented in this figure match those illustrating the multiverse iterations in Figure 3. Solid CIs represent significant correlations, and dashed CIs represent non-significant correlations at *p* = 0.05.

We next examined the impact of mini-multiverse decisions on the associations with the ex-Gaussian parameter estimates. Supplemental Table S5 provides the latent-variable correlations between WMC, TUTs, and the ex-Gaussian parameter estimates across multiverse iterations. These results are visually depicted in Figure 5. Again, the range of estimates across the multiverse was small, +/− 0.07, suggesting high reliability across iterations. The correlations between WMC and the ex-Gaussian parameters were consistent with our main analysis presented earlier: WMC was modestly (and equivalently) correlated with mu and tau and more strongly correlated with sigma. The patterns for TUT rates were also consistent with our main analysis. TUTs were not significantly associated with mu in any iteration of the multiverse. The association with sigma, however, did vary somewhat, and in two cases did not reach significance (*p* > 0.05). However, given that this estimate was the weakest to begin with, it is not surprising that some multiverse paths were not significant. TUT rate’s strong positive correlation with tau was consistent across the multiverse. Our multiverse analyses of the ex-Gaussian parameters, then, found patterns consistent with both the not-best performance rule and the WPR, depending on our measure of cognitive ability.

## 4. Discussion

We reanalyzed data from a large latent-variable study (Kane et al. 2016) to test the robustness of the WPR (or the not-best performance rule; Schubert 2019) across a variety of demanding attention-control tasks. We used two approaches, ranked RT bins and ex-Gaussian estimation, to describe the RT distributions across tasks. In doing so, we assessed latent variables and tested their associations with two cognitive ability constructs, WMC and propensity for TUTs. Our primary findings complement both traditional findings of the WPR and recent meta-analytic claims that cognitive ability is equally predictive of mean and longest RTs, compared to shortest RTs (Schubert 2019). Specifically, WMC showed consistent patterns, at both the task level and latent-variable level, of the not-best performance rule: WMC least strongly predicted subjects’ shortest RTs, but was more strongly—and equally—correlated with their mean and longest RTs; ex-Gaussian analyses showed that WMC correlated at least as strongly with the Gaussian parameters of *sigma* and *mu* as it did with *tau*. TUT rate, on the other hand, showed trends more consistent with the WPR. TUTs were not related to subjects’ shortest RTs (or the *mu* parameter) and were weakly associated with mean RTs; instead, TUT rate correlated most strongly with subjects’ longest RTs (i.e., with both RT Bin 5 and the *tau* parameter). Thus, our results suggest that claims about cognitive ability and worst performance may depend on the ability construct in question. Cognitive abilities that are strongly related to attentional lapses and sustained attention (i.e., propensity for TUTs as assessed by in-task thought probes) may show patterns consistent with the WPR, whereas those that are less strongly related to attentional lapses (i.e., WMC) may show the not-best performance rule.

It is important to note, however, that WMC was not *unrelated* to long RTs (i.e., Bin 5) or *tau*. In fact, the WMC correlations here were of similar magnitude to those of the TUT rate. Instead, WMC correlated with worst *and* mean performance to a similar degree (and best performance to a lesser degree), while TUTs primarily correlated only with worst performance. What might contribute to these different patterns? The association with worst performance is likely driven in part by attention-control ability, which is central to both WMC and TUT propensity. Specifically, the TUT-RT findings are largely supportive of the attentional control theory of WPR. Individuals with poor attentional control, and thus higher likelihood of mind wandering, will experience more attentional lapses than those with better control ability. These occasional attentional lapses result in occasional extremely long RTs that are reflected in the tail of that individuals RT distribution (i.e., *tau* and the slowest RT bin). However, WMC and TUTs are multidetermined constructs, and so combinations of other processes likely also contribute to their associations with RT variables. There are likely many cognitive processes (executive and otherwise) that are associated with WMC, but not TUTs, that also contribute to average RT—such as stimulus-response binding (Wilhelm and Oberauer 2006), speed-accuracy trade-off (Unsworth and Engle 2008), working memory load (Shahar et al. 2014), encoding ability (Unsworth and Spillers 2010), and evidence-accumulation processes (Schmiedek et al. 2007)—and variation in these additional processes contribute to the not-best performance rule pattern for WMC. Thus, the processes that contribute to performance on fast and average RT trials seem to overlap more with WMC processes (and executive processes related to WMC) than with TUT-related processes (Kovacs and Conway 2016).

A methodological issue that arises when assessing the WPR (or any RT or performance phenomenon in psychological science) is how to treat outlier trials and outlying subjects. As noted in the introduction, reporting of such outlier treatment was scarce in the articles included in Schubert’s (2019) meta-analysis of the WPR. This is unfortunate. Bakker and Wicherts (2014) investigated whether simply reporting the removal of outliers was related to weaker evidence in a set of RT studies. Although they found no difference in the strength of evidence between studies that did versus did not report outliers, they did find that there were issues in reporting and suggested there was a common failure to report exclusions or missing data. Bakker and Wicherts argued for greater transparency in reporting of outliers and statistical analyses, and we agree (see also Leys et al. 2019 for a discussion on how to identify and handle outliers in a study).

To explicitly probe the issue of outlier treatment—which prior WPR studies have not considered systematically—we created a mini-multiverse of outlier treatments at both trial and subject levels that are common to the literature (including no treatment). We then re-ran our primary confirmatory factor analyses across these iterations to investigate whether they altered associations between cognitive-ability constructs and aspects of the RT distributions. They did not. That is, the results of our primary analyses replicated across multiverse iterations. Thus, in a study that collects RTs across multiple tasks per subject, and does so for hundreds of subjects, outlier treatment does not significantly affect the assessment of worst performance and individual differences therein. Our multiverse findings cannot say whether outlier decisions are equally irrelevant to conclusions drawn from smaller-N studies using single tasks.

We must acknowledge the study’s limitations, however. First, although we analyzed RTs from only non-conflict trials from six tasks, all the tasks presented some conflict trials, thus creating an “attention-control” context; our findings thus might not generalize to simple or choice RT tasks without conflict trials included. Second, although our RT tasks created an attention-control context, they did not impose significant memory demands. Prior work suggests that such memory demands (i.e., more choices in choice-RT tasks, or arbitrary response mappings) may make the WPR more apparent (Meiran and Shahar 2018; Shahar et al. 2014). For example, Rammsayer and Troche (2016) found a stronger link between WPR and psychometric *g* in 1- and 2-bit versions of the Hick task, compared to the simpler 0-bit version. More complex tasks, such as problem-solving tasks, might also elicit stronger WPR patterns than the not-best performance rule patterns (Kranzler 1992; Ratcliff et al. 2010); at the same time, the more complex a task becomes, the more executive processes may become involved in successful performance, which might yield stronger evidence for the not-best performance rule. Whether one finds evidence for the WPR or the not-best performance rule might therefore vary with both the nature of the cognitive ability construct and the cognitive demands of the RT tasks. An additional limitation of this study is that our assessment of sustained attention ability relied solely on self-reported TUTs. Although these reports have generally been found to be valid indicators of one’s propensity (and, presumably, ability) to sustain attention, they are not pure indicators of ability. Future WPR research should therefore also consider assessing objective performance measures of sustained attention ability, such as RT variability, vigilance decrements, or even pupil size, rather than solely relying on self-report measures (i.e., TUT reports) to test whether the WPR versus the not-best performance rule patterns reported here are also obtained.

## Figures and Tables

**Table 1:** Descriptive statistics for ranked-bin measures for each reaction time task.

Variable	Mean	SD	Min	Max	Skew	Kurtosis
SART Bin 1	337	72	213	639	0.726	0.562
SART Bin 2	421	95	237	823	0.506	0.202
SART Bin 3	491	107	258	979	0.265	0.177
SART Bin 4	576	122	279	1048	0.367	1.002
SART Bin 5	781	182	326	1419	0.870	1.412
Letter Flanker Bin 1	437	59	292	627	0.608	0.417
Letter Flanker Bin 2	498	75	339	773	0.651	0.372
Letter Flanker Bin 3	547	91	367	864	0.778	0.574
Letter Flanker Bin 4	611	118	405	1028	0.982	1.157
Letter Flanker Bin 5	778	202	450	1488	1.168	1.467
Arrow Flanker Bin 1	389	37	260	527	0.306	0.486
Arrow Flanker Bin 2	437	45	311	584	0.552	0.195
Arrow Flanker Bin 3	471	53	343	669	0.691	0.476
Arrow Flanker Bin 4	515	67	373	750	0.776	0.536
Arrow Flanker Bin 5	636	113	427	1048	0.949	0.744
Circle Flanker Bin 1	426	46	293	595	0.668	0.930
Circle Flanker Bin 2	489	57	351	699	0.629	0.516
Circle Flanker Bin 3	536	69	389	799	0.776	1.010
Circle Flanker Bin 4	600	96	421	941	1.131	1.794
Circle Flanker Bin 5	768	180	466	1360	1.339	1.938
Number Stroop Bin 1	411	40	309	557	0.590	0.940
Number Stroop Bin 2	478	47	366	658	0.490	0.831
Number Stroop Bin 3	523	53	405	724	0.480	0.687
Number Stroop Bin 4	574	64	441	824	0.648	0.790
Number Stroop Bin 5	716	125	502	1167	1.228	1.799
Spatial Stroop Bin 1	516	95	293	880	1.013	1.507
Spatial Stroop Bin 2	596	118	382	1010	1.151	1.716
Spatial Stroop Bin 3	661	139	410	1133	1.216	1.714
Spatial Stroop Bin 4	751	179	432	1333	1.334	1.900
Spatial Stroop Bin 5	991	307	514	1955	1.408	1.765

Note. SART = Sustained Attention to Response Task. Bin 1 = subjects’ fastest quintile of RTs; Bin 5 = subjects’ slowest quintile of RTs. Note. SART = Sustained Attention to Response Task. Bin 1 = subjects’ fastest quintile of RTs; Bin 5 = subjects’ slowest quintile of RTs.

**Table 2:** Hierarchical regressions examining the interaction between cognitive predictors and bin each task in predicting RT.

	Model 1 (WMC)		Model 2 (TUTs)	
**SART**	*B (SE)*	*ß*	*B (SE)*	*ß*
Bin	104.655 (1.743)	0.759 ***	104.655 (1.743)	0.759 ***
WMC	−3.677 (7.795)	−0.009		
Bin X WMC	−20.330 (3.556)	−0.169 ***		
TUT			−5.470 (9.427)	−0.011
Bin X TUT			24.743 (4.299)	0.171 ***
**Letter Flanker**				
Bin	79.654 (1.857)	0.665 ***	79.724 (1.824)	0.759 ***
WMC	−31.405 (7.331)	−0.089 ***		
Bin X WMC	−9.604 (3.851)	−0.090 *		
TUT			50.355 (8.434)	0.123 ***
Bin X TUT			20.369 (4.403)	0.165 ***
**Arrow Flanker**	*B (SE)*	*ß*	*B (SE)*	*ß*
Bin	57.324 (1.030)	0.748 ***	57.268 (1.044)	0.747 ***
WMC	−26.804 (4.547)	−0.120 ***		
Bin X WMC	−9.373 (2.115)	−0.139 *		
TUT			17.039 (5.480)	0.064 **
Bin X TUT			8.411 (2.571)	0.104 **
**Circle Flanker**				
Bin	80.380 (1.594)	0.714 ***	80.441 (1.606)	0.714 ***
WMC	−47.011 (6.587)	−0.146 ***		
Bin X WMC	−15.938 (3.220)	−0.169 ***		
TUT			46.040 (7.929)	0.119 ***
Bin X TUT			19.843 (3.890)	0.170 ***
**Number Stroop**				
Bin	70.970 (1.098)	0.795 ***	70.998 (1.102)	0.795 ***
WMC	−32.507 (5.342)	−0.125 ***		
Bin X WMC	−12.222 (2.261)	−0.156 ***		
TUT			32.592 (6.274)	0.107 ***
Bin X TUT			16.167 (2.661)	0.176 ***
**Spatial Stroop**				
Bin	112.604 (2.980)	0.616 ***	112.817 (3.012)	0.618 ***
WMC	−59.276 (10.940)	−0.113 ***		
Bin X WMC	−21.361 (6.060)	−0.139 ***		
TUT			13.043 (13.129)	0.021
Bin X TUT			25.725 (7.301)	0.136 ***

Note. SART = Sustained Attention to Response Task. * *p* < 0.05; ** *p* < 0.01; *** *p*< 0.001.

**Table 3:** Hierarchical regressions of WMC regressed on Bins 1, 3, and 5, for each task.

	Model 1		Model 2		Model 3	
**SART**	*B (SE)*	*ß*	*B (SE)*	*ß*	*B (SE)*	*ß*
Bin 1	0.001 (0.000)	0.159 ***	0.003 (0.001)	0.394 ***	0.002 (0.001)	0.220 *
Bin 3			−0.001 (0.000)	−0.276 ***	0.000 (0.000)	0.018
Bin 5					−0.001 (0.000)	−0.247 *
R^2^	0.025		0.047		0.079	
∆*R*^2^			0.022		0.032	
**Letter Flanker**						
Bin 1	−0.001 (0.000)	−0.105 *	0.002 (0.001)	0.244 *	0.003 (0.001)	0.329 **
Bin 3			−0.002 (0.001)	−0.381 ***	−0.003 (0.001)	−0.619 ***
Bin 5					0.000 (0.000)	0.182 ^
*R* ^2^	0.011		0.034		0.041	
∆*R*^2^			0.022		0.007	
**Arrow Flanker**						
Bin 1	−0.002 (0.001)	−0.138 **	0.003 (0.001)	0.217 *	0.003 (0.001)	0.202 *
Bin 3			−0.004 (0.001)	−0.407 ***	−0.003 (0.001)	−0.356 *
Bin 5					−0.000 (0.000)	−0.041
*R* ^2^	0.019		0.058		0.059	
∆*R*^2^			0.039		0.001	
**Circle Flanker**						
Bin 1	−0.002 (0.000)	−0.230 ***	0.001 (0.001)	0.049	0.001 (0.001)	0.048
Bin 3			−0.002 (0.001)	−0.317 ***	−0.002 (0.001)	−0.315 *
Bin 5					−0.000 (0.000)	−0.002
*R* ^2^	0.053		0.076		0.076	
∆*R*^2^			0.023		0.000	
**Number Stroop**						
Bin 1	−0.002 (0.001)	−0.135 **	0.005 (0.001)	0.410 ***	0.006 (0.001)	0.457 ***
Bin 3			−0.006 (0.001)	−0.621 ***	−0.007 (0.001)	−0.730 ***
Bin 5					0.000 (0.000)	0.083
*R* ^2^	0.018		0.106		0.108	
∆*R*^2^			0.088		0.002	
**Spatial Stroop**	*B (SE)*	*ß*	*B (SE)*	*ß*	*B (SE)*	*ß*
Bin 1	−0.001 (0.000)	−0.128 **	0.001 (0.001)	0.149	0.000 (0.001)	0.092
Bin 3			−0.001 (0.000)	−0.300 *	−0.001 (0.001)	−0.185
Bin 5					−0.000 (0.000)	−0.074
*R* ^2^	0.016		0.030		0.031	
∆*R*^2^			0.014		0.001	

Note. SART = Sustained Attention to Response Task. ^ *p* < 0.10; * *p* < 0.05; ** *p* < 0.01; *** *p* < 0.001.

**Table 4:** Hierarchical regressions of TUTs regressed on Bins 1, 3, and 5, for each task.

	Model 1		Model 2		Model 3	
**SART**	*B (SE)*	*ß*	*B (SE)*	*ß*	*B (SE)*	*ß*
Bin 1	−0.001 (0.000)	−0.210 ***	−0.003 (0.000)	−0.453 ***	−0.002 (0.001)	−0.291 **
Bin 3			0.001 (0.000)	0.287 ***	0.000 (0.000)	0.012
Bin 5					0.001 (0.000)	0.231 ***
R^2^	0.044		0.067		0.094	
∆*R*^2^			0.020		0.027	
**Letter Flanker**						
Bin 1	0.001 (0.000)	0.136 **	−0.001 (0.001)	−0.191 ^	−0.001 (0.001)	−0.092
Bin 3			0.002 (0.001)	0.358 **	0.000 (0.001)	0.080
Bin 5					0.000 (0.000)	0.213 *
*R* ^2^	0.019		0.039		0.048	
∆*R*^2^			0.020		0.009	
**Arrow Flanker**						
Bin 1	0.000 (0.001)	0.031	−0.003 (0.001)	−0.241 **	−0.002 (0.001)	−0.180 ^
Bin 3			0.002 (0.001)	0.312 ***	0.001 (0.001)	0.112
Bin 5					0.001 (0.000)	0.165
*R* ^2^	0.001		0.024		0.029	
∆*R*^2^			0.023		0.005	
**Circle Flanker**						
Bin 1	0.001 (0.000)	0.155 ***	−0.001 (0.001)	−0.067	0.000 (0.001)	0.031
Bin 3			0.001 (0.001)	0.252 **	−0.000 (0.001)	−0.018
Bin 5					0.000 (0.000)	0.220 *
*R* ^2^	0.024		0.038		0.051	
∆*R*^2^			0.014		0.013	
**Number Stroop**	*B (SE)*	*ß*	*B (SE)*	*ß*	*B (SE)*	*ß*
Bin 1	0.001 (0.00)	0.089 ^	−0.003 (0.001)	−0.295 **	−0.001 (0.001)	−0.101
Bin 3			0.003 (0.001)	0.437 ***	−0.000 (0.001)	−0.020
Bin 5					0.001 (0.000)	0.345 ***
*R* ^2^	0.008		0.052		0.080	
∆*R*^2^			0.044		0.028	
**Spatial Stroop**						
Bin 1	−0.000 (0.000)	− 0.107 *	−0.003 (0.001)	−0.672 ***	−0.002 (0.001)	−0.513 ***
Bin 3			0.002 (0.000)	0.612 ***	0.001 (0.001)	0.286
Bin 5					0.000 (0.000)	0.209 ^
*R* ^2^	0.011		0.068		0.075	
∆*R*^2^			0.057		0.007	

Note. SART = Sustained Attention to Response Task. ^ *p* < 0.10; * *p* < 0.05; ** *p* < 0.01; *** *p* < 0.001.

**Table 5:** Descriptive statistics for ex-Gaussian measures.

Variable	Mean	SD	Min	Max	Skew	Kurtosis
SART *Mu*	447	49	330	646	0.678	0.927
SART *Sigma*	49	19	0	119	0.309	0.571
SART *Tau*	114	61	1	303	1.263	1.704
Letter Flanker *Mu*	376	108	200	871	0.465	0.109
Letter Flanker *Sigma*	69	41	0	252	0.758	0.460
Letter Flanker *Tau*	144	71	3	386	1.147	1.670
Arrow Flanker *Mu*	446	42	356	608	0.446	0.639
Arrow Flanker *Sigma*	58	14	22	108	0.523	0.456
Arrow Flanker *Tau*	94	42	5	237	1.335	1.801
Circle Flanker *Mu*	407	39	309	533	0.506	0.300
Circle Flanker *Sigma*	39	15	0	91	0.917	1.366
Circle Flanker *Tau*	82	36	3	203	0.968	0.796
Number Stroop *Mu*	534	105	329	917	1.046	1.593
Number Stroop *Sigma*	58	30	0	167	1.024	1.852
Number Stroop *Tau*	165	98	3	486	1.353	1.816
Spatial Stroop *Mu*	456	65	310	702	0.645	0.444
Spatial Stroop *Sigma*	47	21	0	127	0.730	0.879
Spatial Stroop *Tau*	115	64	2	345	1.210	1.851

Note. SART = Sustained Attention to Response Task.

## Supplementary Materials

The following are available online at https://www.mdpi.com/2079-3200/8/2/25/s1, Figure S1: RT predicted by WMC x Bin for each task. Figure S2: RT predicted by TUT x Bin for each task. Table S1: Zero-order correlations between cognitive predictors and ranked-bin variables. Table S2: Zero-order correlations between cognitive predictors and ex-Gaussian variables. Table S3: Standardized factor loadings (with standard errors) for latent-variable models. Table S4: Multiverse correlation matrix between WMC, TUTs, and ranked-bin latent variables. Table S5: Multiverse correlation matrix between WMC, TUTs, and ex-Gaussian latent variables.
