# Supplementary material for: The Worst Performance Rule, or the Not-Best Performance Rule? Latent-Variable Analyses of Working Memory Capacity, Mind-Wandering Propensity, and Reaction Time
Source: J Intell. 2020 Jun 2;8(2):25. doi: 10.3390/jintelligence8020025 (PMC7713012; doi:10.3390/jintelligence8020025)
Supplement: Supplementary file 1 [file jintelligence-08-00025-s001.pdf]

Supplemental Table 1. Zero-order correlations between cognitive predictors and ranked bin variables.

Variable	1	2	3	4	5	6	7	8	9	10	11	12	13	14	15	16
1. OPERSPAN	1															
2. READSPAN	0.58	1														
3. SYMMSPAN	0.40	0.38	1													
4. ROTSPAN	0.44	0.32	0.54	1												
5. RUNNSPAN	0.45	0.37	0.27	0.20	1											
6. COUNTERS	0.36	0.23	0.37	0.29	0.39	1										
7. SART-TUT	-0.01	-0.14	-0.08	0.00	-0.05	-0.06	1									
8. NUMS-TUT	-0.03	-0.11	-0.03	-0.03	-0.12	-0.02	0.45	1								
9. ARRO-TUT	0.01	-0.08	-0.03	0.02	-0.11	-0.04	0.43	0.67	1							
10. LETT-TUT	0.08	0.01	-0.09	-0.01	0.04	-0.02	0.52	0.32	0.36	1						
11.2BAC-TUT	-0.05	-0.09	-0.06	-0.14	-0.20	-0.12	0.39	0.43	0.41	0.33	1					
12. SART Bin1	0.11	0.10	0.11	0.10	0.11	0.09	-0.11	-0.16	-0.13	-0.08	-0.24	1				
13. SART Bin3	0.06	0.08	0.03	0.03	0.03	-0.01	-0.01	-0.12	-0.08	-0.02	-0.11	0.85	1			
14. SART Bin5	-0.06	-0.04	-0.11	-0.04	-0.18	-0.20	0.20	0.06	0.07	0.07	0.13	0.32	0.6	1		
15. LETT Bin1	-0.03	0.03	-0.15	-0.10	-0.02	-0.16	0.13	0.08	0.08	0.18	0.09	0.11	0.16	0.25	1	
16. LETT Bin3	-0.07	0.00	-0.19	-0.14	-0.07	-0.21	0.17	0.11	0.09	0.24	0.13	0.04	0.13	0.32	0.92	1
17. LETT Bin5	-0.03	-0.03	-0.16	-0.12	-0.08	-0.18	0.21	0.13	0.09	0.28	0.19	-0.01	0.10	0.32	0.74	0.89
18. ARRO Bin1	-0.08	-0.03	-0.14	-0.08	-0.13	-0.12	-0.05	0.04	0.07	0.00	0.03	0.15	0.15	0.15	0.43	0.41
19. ARRO Bin3	-0.12	-0.06	-0.17	-0.13	-0.16	-0.24	0.01	0.09	0.10	0.05	0.08	0.02	0.06	0.20	0.53	0.54
20. ARRO Bin5	-0.11	-0.05	-0.14	-0.14	-0.17	-0.25	0.01	0.13	0.15	0.07	0.11	-0.05	0.01	0.19	0.43	0.47
21. CIRCFLNK Bin1	-0.14	-0.10	-0.18	-0.17	-0.16	-0.19	0.11	0.12	0.13	0.13	0.08	0.13	0.21	0.32	0.49	0.48
22. CIRCFLNK Bin3	-0.17	-0.12	-0.20	-0.21	-0.17	-0.26	0.13	0.15	0.16	0.15	0.11	0.08	0.18	0.34	0.5	0.54
23. CIRCFLNK Bin5	-0.11	-0.09	-0.13	-0.17	-0.15	-0.29	0.15	0.19	0.19	0.14	0.15	0.03	0.15	0.32	0.37	0.43
24. N-STROOP Bin1	-0.08	-0.06	-0.13	-0.13	-0.09	-0.10	0.04	0.10	0.09	0.02	0.04	0.15	0.20	0.25	0.45	0.39
25. N-STROOP Bin3	-0.16	-0.10	-0.22	-0.21	-0.16	-0.22	0.10	0.18	0.12	0.09	0.12	0.02	0.12	0.31	0.48	0.47
26. N-STROOP Bin5	-0.12	-0.06	-0.22	-0.17	-0.16	-0.23	0.15	0.26	0.17	0.18	0.19	-0.07	0.05	0.32	0.37	0.41
27. S-STROOP Bin1	-0.08	-0.04	-0.19	-0.07	-0.06	-0.14	-0.04	-0.10	-0.04	-0.05	-0.16	0.20	0.20	0.12	0.39	0.36
28. S-STROOP Bin3	-0.09	-0.04	-0.17	-0.10	-0.10	-0.16	0.03	-0.02	0.01	0.02	-0.05	0.14	0.17	0.16	0.39	0.40
29. S-STROOP Bin5	-0.10	-0.04	-0.13	-0.10	-0.13	-0.18	0.09	0.04	0.05	0.07	0.07	0.04	0.10	0.21	0.32	0.35

Variable	17	18	19	20	21	22	23	24	25	26	27	28
1. OPERSPAN												
2. READSPAN												
3. SYMMSPAN												
4. ROTSPAN												
5. RUNNSPAN												
6. COUNTERS												
7. SART-TUT												
8. NUMS-TUT												
9. ARRO-TUT												
10. LETT-TUT												
11.2BAC-TUT												
12. SART Bin1												
13. SART Bin3												
14. SART Bin5												
15. LETT Bin1												
16. LETT Bin3												
17. LETT Bin5	1											
18. ARRO Bin1	0.30	1										
19. ARRO Bin3	0.43	0.87	1									
20. ARRO Bin5	0.42	0.69	0.89	1								
21. CIRCFLNK Bin1	0.42	0.56	0.59	0.50	1							
22. CIRCFLNK Bin3	0.49	0.51	0.61	0.58	0.88	1						
23. CIRCFLNK Bin5	0.44	0.33	0.48	0.54	0.65	0.85	1					
24. N-STROOP Bin1	0.32	0.57	0.57	0.43	0.57	0.53	0.32	1				
25. N-STROOP Bin3	0.41	0.52	0.62	0.53	0.60	0.64	0.49	0.88	1			
26. N-STROOP Bin5	0.41	0.29	0.44	0.45	0.46	0.57	0.54	0.60	0.84	1		
27. S-STROOP Bin1	0.26	0.44	0.45	0.35	0.37	0.32	0.19	0.39	0.36	0.19	1	
28. S-STROOP Bin3	0.33	0.42	0.47	0.41	0.38	0.37	0.29	0.39	0.40	0.29	0.92	1
29. S-STROOP Bin5	0.35	0.29	0.39	0.40	0.31	0.35	0.35	0.31	0.38	0.36	0.69	0.87

Supplemental Table 2. Zero order correlations between cognitive predictors and ex-Gaussian variables.

Variable	1	2	3	4	5	6	7	8	9	10	11	12	13	14	15	16
1. OPERSPAN	1.00															
2. READSPAN	0.58	1.00														
3. SYMMSPAN	0.40	0.38	1.00													
4. ROTSPAN	0.44	0.32	0.54	1.00												
5. RUNNSPAN	0.45	0.37	0.27	0.20	1.00											
6. COUNTERS	0.36	0.23	0.37	0.29	0.39	1.00										
7. SART-TUT	-0.01	-0.14	-0.08	0.00	-0.05	-0.06	1.00									
8. NUMS-TUT	-0.03	-0.11	-0.03	-0.03	-0.12	-0.02	0.45	1.00								
9. ARRO-TUT	0.01	-0.08	-0.03	0.02	-0.11	-0.04	0.43	0.67	1.00							
10. LETT-TUT	0.08	0.01	-0.09	-0.01	0.04	-0.02	0.52	0.32	0.36	1.00						
11.2BAC-TUT	-0.05	-0.09	-0.06	-0.14	-0.20	-0.12	0.39	0.43	0.41	0.33	1.00					
12. SART Mu	0.10	0.11	0.10	0.08	0.13	0.11	-0.13	-0.18	-0.15	-0.09	-0.21	1.00				
13. SART Sigma	0.02	0.05	0.03	0.00	0.06	0.04	-0.04	-0.12	-0.09	-0.01	-0.06	0.82	1.00			
14. SART Tau	-0.11	-0.11	-0.16	-0.08	-0.24	-0.26	0.27	0.17	0.16	0.13	0.24	-0.44	-0.29	1.00		
15. LETT Mu	-0.08	0.02	-0.16	-0.13	-0.02	-0.17	0.09	0.07	0.06	0.14	0.06	0.04	0.03	0.20	1.00	
16. LETT Sigma	-0.19	-0.06	-0.19	-0.21	-0.09	-0.17	0.03	0.04	-0.01	0.10	0.03	-0.03	0.02	0.14	0.70	1.00
17. LETT Tau	0.00	-0.05	-0.12	-0.08	-0.09	-0.15	0.20	0.11	0.09	0.27	0.19	-0.08	0.01	0.32	0.25	0.07
18. ARRO Mu	-0.10	-0.04	-0.15	-0.08	-0.11	-0.17	-0.01	0.02	0.04	0.01	0.03	0.06	0.05	0.11	0.50	0.33
19. ARRO Sigma	-0.11	-0.07	-0.14	-0.13	-0.07	-0.25	0.14	0.04	0.02	0.09	0.11	-0.17	-0.08	0.26	0.42	0.34
20. ARRO Tau	-0.08	-0.04	-0.11	-0.13	-0.15	-0.21	0.02	0.15	0.16	0.06	0.12	-0.14	-0.11	0.25	0.21	0.16
21. CIRCFLNK Mu	-0.18	-0.11	-0.19	-0.18	-0.13	-0.15	0.07	0.02	0.06	0.11	0.04	0.09	0.11	0.19	0.46	0.29
22. CIRCFLNK Sigma	-0.15	-0.10	-0.13	-0.17	-0.05	-0.12	0.05	-0.04	-0.04	0.04	0.01	-0.02	0.02	0.13	0.27	0.31
23. CIRCFLNK Tau	-0.05	-0.06	-0.08	-0.12	-0.12	-0.27	0.14	0.21	0.19	0.11	0.14	-0.01	0.06	0.26	0.19	0.16
24. N-STROOP Mu	-0.13	-0.10	-0.15	-0.17	-0.10	-0.12	0.02	0.04	0.05	-0.01	0.02	0.10	0.09	0.10	0.41	0.23
25. N-STROOP Sigma	-0.22	-0.14	-0.21	-0.22	-0.17	-0.24	0.08	0.07	0.05	0.06	0.10	-0.20	-0.14	0.23	0.25	0.27
26. N-STROOP Tau	-0.07	-0.02	-0.18	-0.11	-0.12	-0.21	0.17	0.27	0.17	0.19	0.20	-0.15	-0.04	0.37	0.17	0.10
27. S-STROOP Mu	-0.08	-0.04	-0.18	-0.07	-0.04	-0.11	-0.04	-0.10	-0.04	-0.05	-0.17	0.22	0.17	-0.08	0.34	0.19
28. S-STROOP Sigma	-0.07	-0.03	-0.09	-0.10	-0.06	-0.06	0.07	0.04	0.07	0.04	0.04	0.06	0.09	0.02	0.18	0.17
29. S-STROOP Tau	-0.07	-0.02	-0.08	-0.10	-0.14	-0.16	0.11	0.10	0.07	0.09	0.15	-0.07	-0.02	0.21	0.17	0.12

Variable	17	18	19	20	21	22	23	24	25	26	27	28
1. OPERSPAN												
2. READSPAN												
3. SYMMSPAN												
4. ROTSPAN												
5. RUNNSPAN												
6. COUNTERS												
7. SART-TUT												
8. NUMS-TUT												
9. ARRO-TUT												
10. LETT-TUT												
11.2BAC-TUT												
12. SART Mu												
13. SART Sigma												
14. SART Tau												
15. LETT Mu												
16. LETT Sigma												
17. LETT Tau	1.00											
18. ARRO Mu	0.49	1.00										
19. ARRO Sigma	0.25	0.16	1.00									
20. ARRO Tau	0.58	0.35	0.23	1.00								
21. CIRCFLNK Mu	0.22	0.31	0.13	0.53	1.00							
22. CIRCFLNK Sigma	0.15	0.16	0.46	0.11	0.00	1.00						
23. CIRCFLNK Tau	0.63	0.34	0.22	0.59	0.28	0.10	1.00					
24. N-STROOP Mu	0.28	0.43	0.26	0.28	0.27	0.24	0.47	1.00				
25. N-STROOP Sigma	0.11	0.24	0.35	0.18	0.13	0.48	0.07	0.18	1.00			
26. N-STROOP Tau	0.43	0.12	0.19	0.36	0.13	0.07	0.41	0.13	-0.02	1.00		
27. S-STROOP Mu	0.21	0.06	0.24	0.24	0.15	0.17	0.25	0.17	0.04	0.65	1.00	
28. S-STROOP Sigma	0.15	0.21	0.32	0.10	0.03	0.32	0.15	0.17	0.34	0.20	0.15	1.00
29. S-STROOP Tau	1.00	0.49	0.25	0.58	0.22	0.15	0.63	0.28	0.11	0.43	0.21	0.15

Supplemental Table 3. Standardized Factor Loadings (With Standard Errors) for Latent Variable Models

Construct and Measure	Structural Models	
	Ranked Bin CFA	Ex-Gaussian CFA
Working Memory Capacity		
OPERSPAN	.66 (.05)	.66 (.05)
READSPAN	.52 (.05)	.52 (.05)
SYMSPAN	.60 (.05)	.60 (.05)
ROTSPAN	.53 (.06)	.53 (.06)
RUNSPAN	.59 (.05)	.59 (.05)
COUNTERS	.61 (.04)	.61 (.04)
TUTs		
SART	.64 (.06)	.64 (.06)
LETTER FLANKER	.50 (.06)	.50 (.06)
ARROW FLANKER	.66 (.05)	.66 (.05)
N-STROOP	.69 (.05)	.69 (.05)
N-BACK	.63 (.05)	.63 (.05)
Bin 1		
LETTER FLANKER	.64 (.05)	
CIRCLE FLANKER	.77 (.03)	
ARROW FLANKER	.73 (.04)	
N-STROOP	.79 (.03)	
S-STROOP	.58 (.06)	
SART	.22 (.07)	
Bin 3		
LETTER FLANKER	.64 (.05)	
CIRCLE FLANKER	.81 (.03)	
ARROW FLANKER	.80 (.03)	
N-STROOP	.83 (.03)	
S-STROOP	.59 (.05)	
SART	.30 (.05)	
Bin 5		
LETTER FLANKER	.57 (.05)	
CIRCLE FLANKER	.73 (.04)	

Table 3, continued. Standardized Factor Loadings (With Standard Errors) for Latent Variable Models.

Construct and Measure	Structural Models	
	Ranked Bin CFA	Ex-Gaussian CFA
Bin 5		
ARROW FLANKER	.73 (.04)	
N-STROOP	.76 (.03)	
S-STROOP	.50 (.05)	
SART	.48 (.04)	
Mu		
LETTER FLANKER		.57 (.06)
CIRCLE FLANKER		.77 (.04)
ARROW FLANKER		.82 (.03)
N-STROOP		.80 (.03)
S-STROOP		.56 (.07)
SART		.16 (.06)
Sigma		
LETTER FLANKER		.39 (.10)
CIRCLE FLANKER		.51 (.10)
ARROW FLANKER		.68 (.05)
N-STROOP		.64 (.05)
S-STROOP		.39 (.07)
SART		.21 (.05)
Tau		
LETTER FLANKER		.54 (.06)
CIRCLE FLANKER		.70 (.05)
ARROW FLANKER		.62 (.05)
N-STROOP		.70 (.04)
S-STROOP		.46 (.05)
SART		.41 (.05)

Note. OPERSPAN = operation span; READSPAN = reading span; SYMMSPAN = symmetry span; ROTASPAN = rotation span; RUNNSPAN = running span; COUNTERS = updating counters; N-Stroop = number Stroop; S-Stroop = spatial Stroop; NS = number Stroop task; LF = letter flanker task; AF = arrow flanker task; 2B = 2-back task.

Supplemental Table 4. Multiverse Correlation Matrix between WMC, TUTs, and Ranked Bin Latent Variables

Trials Censored to Mean + 3*IQR Outliers Inc.				
	WMC	TUT	Bin 1	Bin 3
Bin 1	-.295***	.090		
Bin 3	-.402***	.196**	.942***	
Bin 5	-.408***	.326***	.759***	.922***
Trials Censored to Mean + 3*IQR Outliers Removed				
Bin 1	-.272***	.100		
Bin 3	-.382***	.219**	.935***	
Bin 5	-.396***	.340***	.744***	.922***
Trials Censored to Mean + 3*IQR Outliers Censored to Mean + 3*IQR				
Bin 1	-.284***	.099		
Bin 3	-.391***	.218**	.936***	
Bin 5	-.404***	.343***	.742***	.920***
Trials Cut if > Mean + 3*IQR Outliers Inc.				
Bin 1	-.289***	.087		
Bin 3	-.396***	.192**	.943***	
Bin 5	-.415***	.302***	.793***	.946***
Trials Cut if > Mean + 3*IQR Outliers Removed				
Bin 1	-.261***	.103		
Bin 3	-.371***	.218**	.935***	
Bin 5	-.400***	.325***	.780***	.945***
Trials Cut if > Mean + 3*IQR Outliers Censored to Mean + 3*IQR				
Bin 1	-.277***	.098		
Bin 3	-.385***	.214**	.937***	

Bin 5	-.415***	.323***	.780***	.944***
Trials Censored to Mean + 3.5*SD Outliers Inc.				
Bin 1	-.296***	.091		
Bin 3	-.404***	.195**	.943***	
Bin 5	-.399***	.334***	.735***	.902***
Trials Censored to Mean + 3.5*SD Outliers Removed				
Bin 1	-.272***	.102		
Bin 3	-.384***	.220**	.934***	
Bin 5	-.391***	.349***	.719***	.905***

Trials Censored to Mean + 3.5*SD Outliers Censored to Mean + 3*IQR				
	WMC	TUT	Bin 1	Bin 3
Bin 1	-.283***	.101		
Bin 3	-.392***	.219**	.936***	
Bin 5	-.388***	.360***	.707***	.895***
Trials Cut if > Mean + 3.5*SD Outliers Inc.				
Bin 1	-.295***	.089		
Bin 3	-.401***	.192**	.944***	
Bin 5	-.397***	.322***	.767***	.922***
Trials Cut if > Mean + 3.5*SD Outliers Removed				
Bin 1	-.273***	.102		
Bin 3	-.383***	.219**	.935***	
Bin 5	-.388***	.343***	.749***	.924***
Trials Cut if > Mean + 3.5*SD Outliers Censored to Mean + 3*IQR				
Bin 1	-.283***	.099		

Bin 3	-.389***	.216**	.937***	
Bin 5	-.388***	.350***	.743***	.917***
No Trials Removed Outliers Inc.				
Bin 1	-.296***	.092		
Bin 3	-.405***	.196**	.942***	
Bin 5	-.398***	.347***	.722***	.893***
No Trials Removed Outliers Removed				
Bin 1	-.273***	.105		
Bin 3	-.384***	.222**	.935***	
Bin 5	-.397***	.356***	.721***	.906***
No Trials Removed Outliers Censored to Mean + 3*IQR				
Bin 1	-.282***	.102		
Bin 3	-.393***	.221**	.936***	
Bin 5	-.387***	.367***	.698***	.887***

Supplemental Table 5. Multiverse Correlation Matrix between WMC, TUTs, and Ex-Gaussian Latent Variables

Trials Censored to Mean + 3*IQR Outliers Inc.				
	WMC	TUT	Mu	Sigma
Mu	-.309**	.032		
Sigma	-.481***	.167*	.760**	
Tau	-.357***	.395***	.405**	.612***
Trials Censored to Mean + 3*IQR Outliers Removed				
Mu	-.272***	.046		
Sigma	-.473***	.184*	.708***	
Tau	-.349***	.404***	.388***	.561***
Trials Censored to Mean + 3*IQR Outliers Censored to Mean + 3*IQR				
Mu	-.293***	.044		
Sigma	-.483***	.183*	.723***	
Tau	-.352***	.403***	.382***	.579***
Trials Cut if > Mean + 3*IQR Outliers Inc.				
Mu	-.326**	.051		
Sigma	-.496***	.192*	.767***	
Tau	-.360***	.391***	.476***	.706***
Trials Cut if > Mean + 3*IQR Outliers Removed				
Mu	-.290***	.071		
Sigma	-.485***	.212**	.720***	
Tau	-.360***	.404***	.482***	.655***
Trials Cut if > Mean + 3*IQR Outliers Censored to Mean + 3*IQR				
Mu	-.310***	.064		
Sigma	-.497***	.209**	.733***	

Tau	-.360***	.397***	.464***	.676***
Trials Censored to Mean + 3.5*SD Outliers Inc.				
Mu	-.305***	.018		
Sigma	-.487***	.146^	.756**	
Tau	-.340***	.405***	.335**	.528***
Trials Censored to Mean + 3.5*SD Outliers Removed				
Mu	-.266***	.029		
Sigma	-.476***	.162*	.699***	
Tau	-.335***	.392***	.330**	.494***

Trials Censored to Mean + 3.5*SD Outliers Censored to Mean + 3*IQR				
	WMC	TUT	Mu	Sigma
Mu	-.289***	.027		
Sigma	-.489***	.156*	.721***	
Tau	-.339***	.410***	.312**	.499***
Trials Cut if > Mean + 3.5*SD Outliers Inc.				
Mu	-.320**	.019		
Sigma	-.496***	.140^	.765***	
Tau	-.329***	.408***	.370***	.553***
Trials Cut if > Mean + 3.5*SD Outliers Removed				
Mu	-.285***	.031		
Sigma	-.481***	.155*	.713***	
Tau	-.334***	.415***	.366***	.508***
Trials Cut if > Mean + 3.5*SD Outliers Censored to Mean + 3*IQR				
Mu	-.305***	.029		

Sigma	-.496***	.151*	.733***	
Tau	-.328***	.416***	.347***	.517***
No Trials Removed Outliers Inc.				
Mu	-.308**	.023		
Sigma	-.490**	.152^	.759*	
Tau	-.338***	.405***	.351**	.544***
No Trials Removed Outliers Removed				
Mu	-.272***	.036		
Sigma	-.481***	.170*	.706***	
Tau	-.339***	.402***	.340***	.510***
No Trials Removed Outliers Censored to Mean + 3*IQR				
Mu	-.293***	.034		
Sigma	-.496***	.166*	.722***	
Tau	-.337***	.413***	.323***	.512***

Supplemental Figure 1. RT predicted by WMC x Bin for each task.

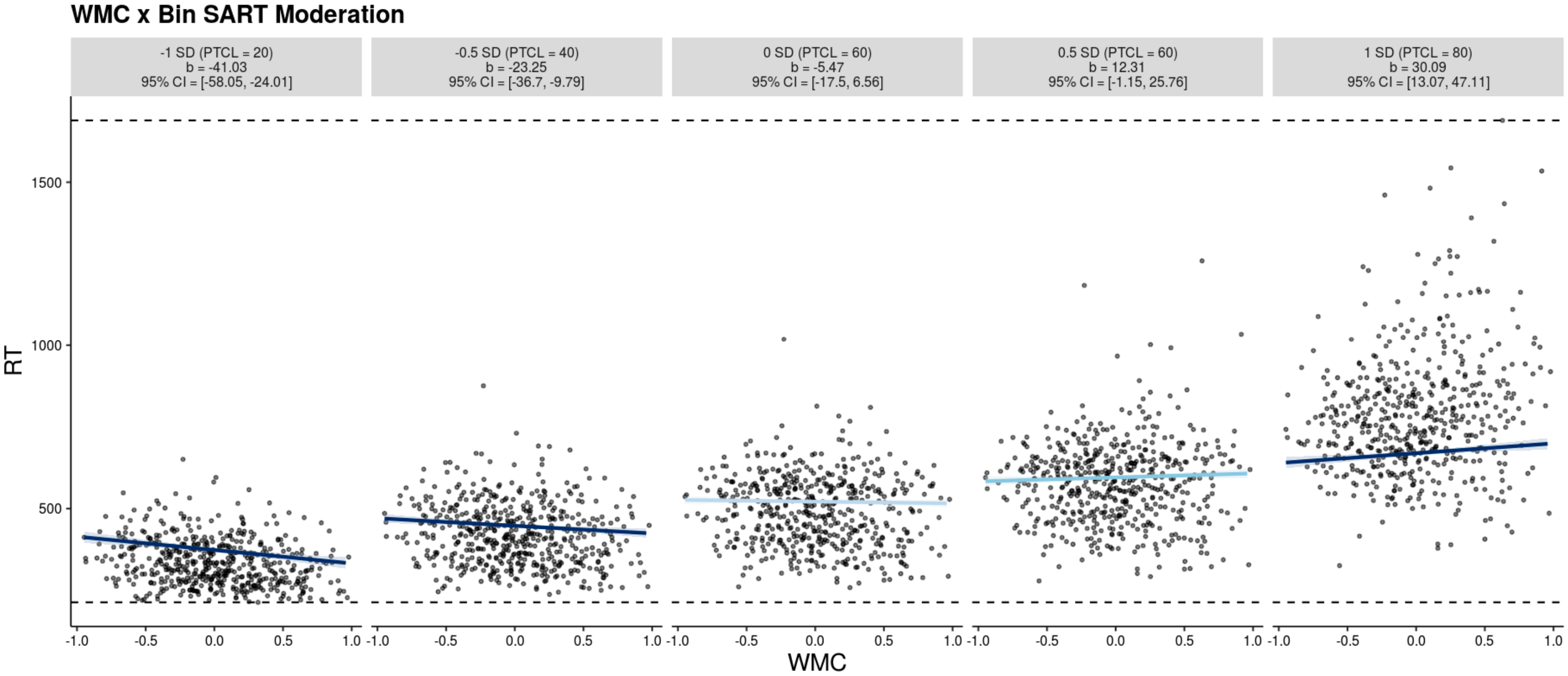

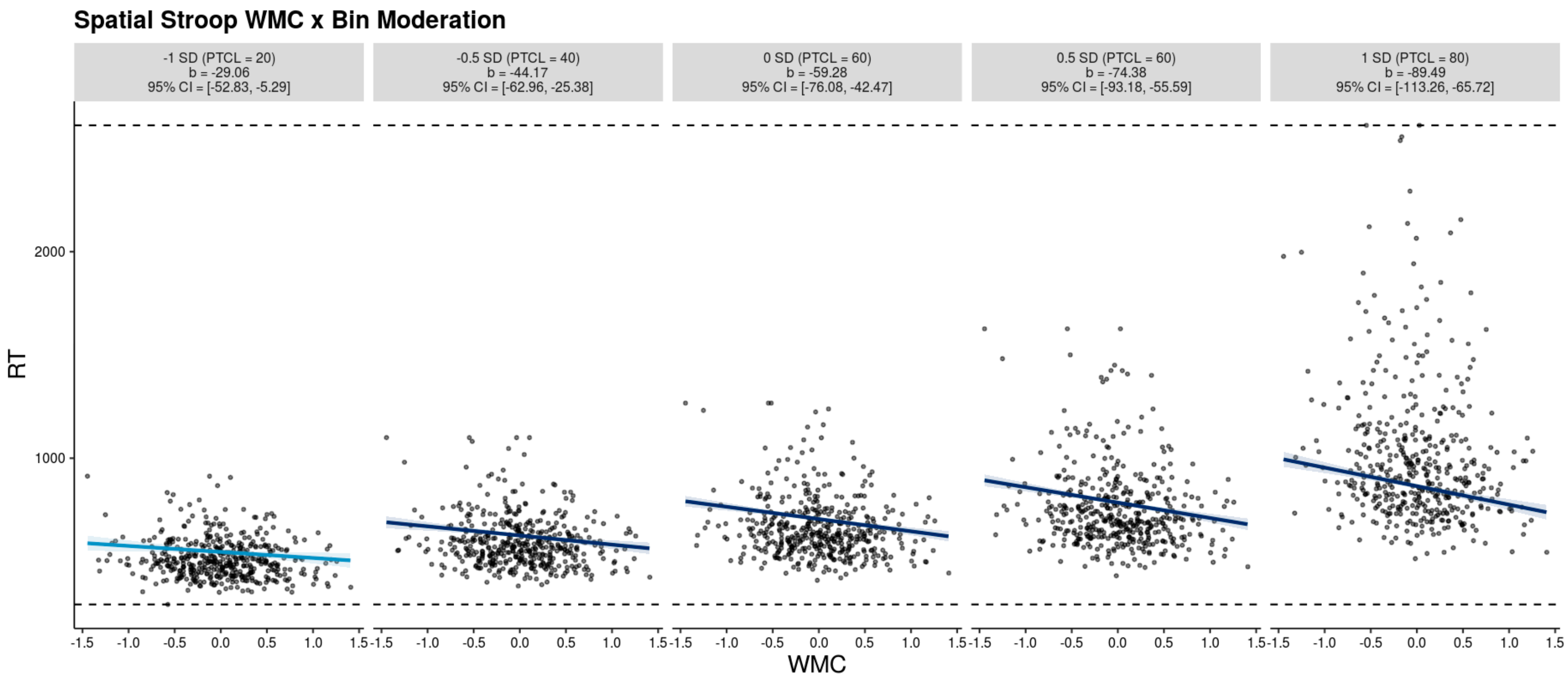
Spatial Stroop WMC x Bin Moderation
-1 SD (PTCL = 20)
b = -29.06
95% CI = [-52.83, -5.29]
-0.5 SD (PTCL = 40)
b = -44.17
95% CI = [-62.96, -25.38]
0 SD (PTCL = 60)
b = -59.28
95% CI = [-76.08, -42.47]
0.5 SD (PTCL = 60)
b = -74.38
95% CI = [-93.18, -55.59]
1 SD (PTCL = 80)
b = -89.49
95% CI = [-113.26, -65.72]
RT
2000
1000
-1.5
-1.0
-0.5
0.0
0.5
1.0
1.5
WMC

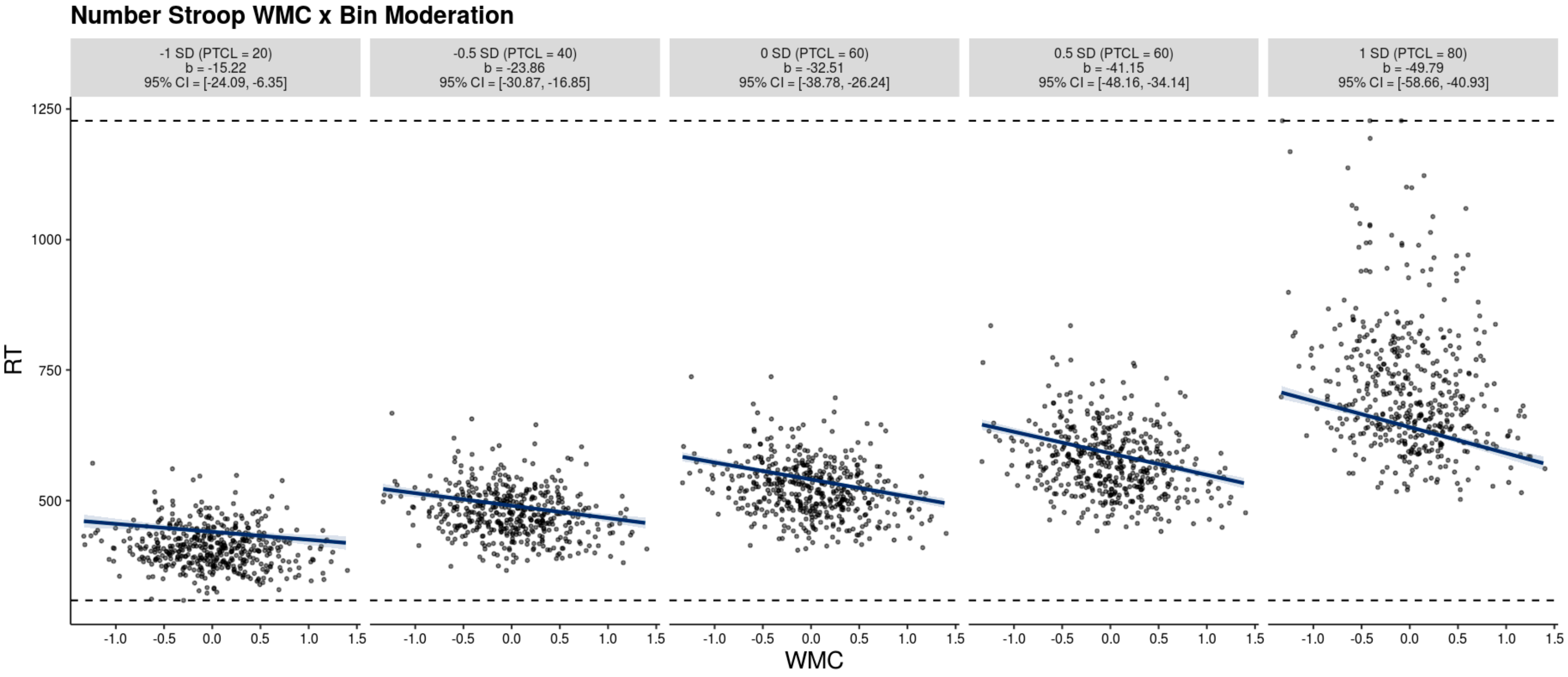

Number Stroop WMC x Bin Moderation
-1 SD (PTCL = 20)
b = -15.22
95% CI = [-24.09, -6.35]
-0.5 SD (PTCL = 40)
b = -23.86
95% CI = [-30.87, -16.85]
0 SD (PTCL = 60)
b = -32.51
95% CI = [-38.78, -26.24]
0.5 SD (PTCL = 60)
b = -41.15
95% CI = [-48.16, -34.14]
1 SD (PTCL = 80)
b = -49.79
95% CI = [-58.66, -40.93]
RT
1250
1000
750
500
-1.0
-0.5
0.0
0.5
1.0
1.5
WMC

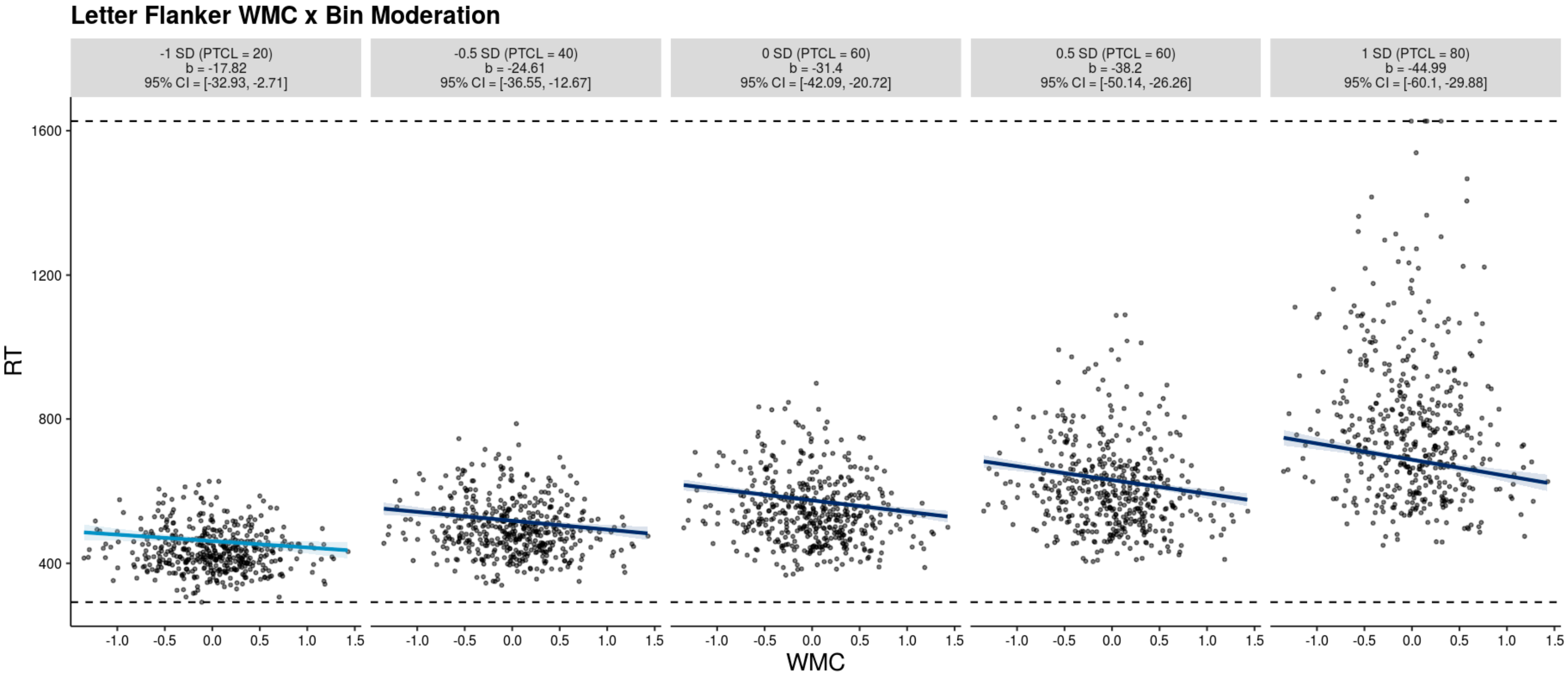
Letter Flanker WMC x Bin Moderation
-1 SD (PTCL = 20)
b = -17.82
95% CI = [-32.93, -2.71]
-0.5 SD (PTCL = 40)
b = -24.61
95% CI = [-36.55, -12.67]
0 SD (PTCL = 60)
b = -31.4
95% CI = [-42.09, -20.72]
0.5 SD (PTCL = 60)
b = -38.2
95% CI = [-50.14, -26.26]
1 SD (PTCL = 80)
b = -44.99
95% CI = [-60.1, -29.88]
RT
1600
1200
800
400
-1.0 -0.5 0.0 0.5 1.0 1.5
WMC

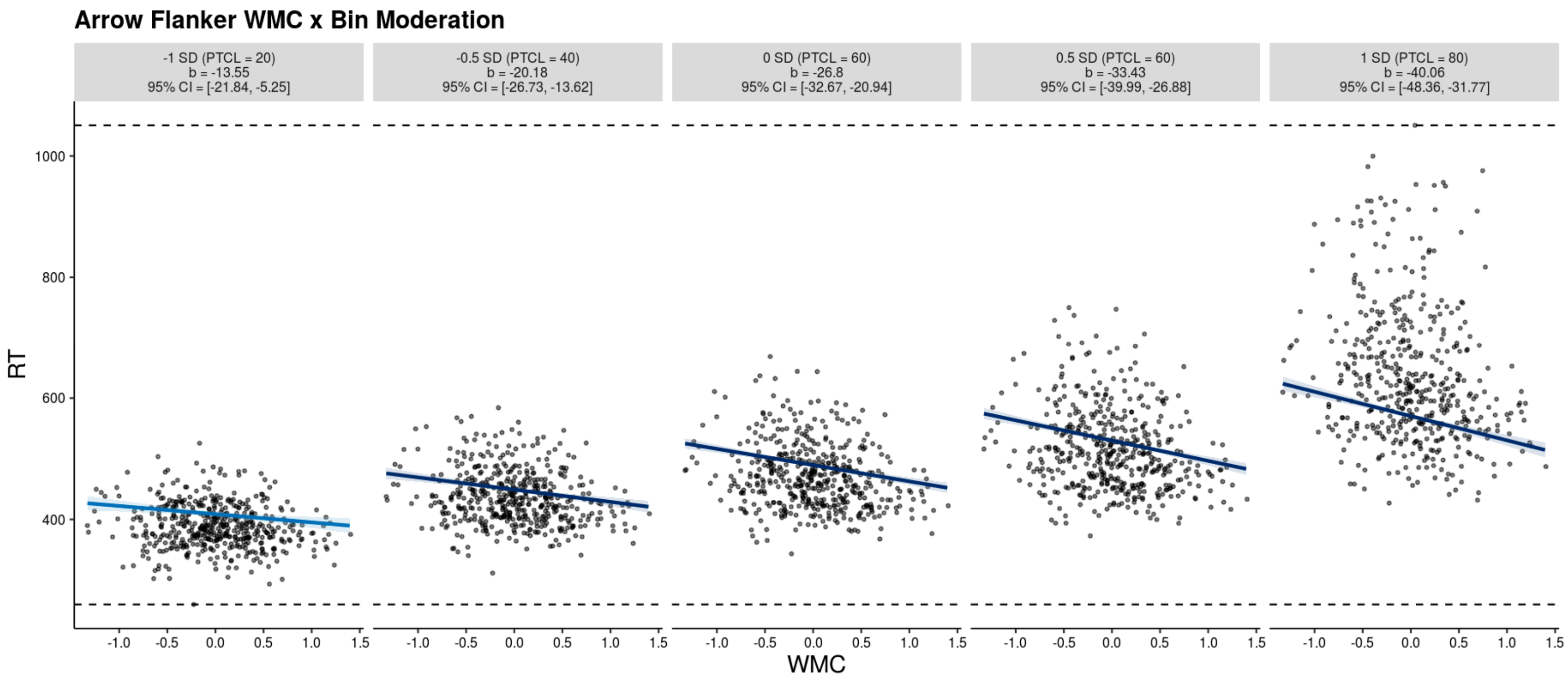

Arrow Flanker WMC x Bin Moderation
-1 SD (PTCL = 20)
b = -13.55
95% CI = [-21.84, -5.25]
-0.5 SD (PTCL = 40)
b = -20.18
95% CI = [-26.73, -13.62]
0 SD (PTCL = 60)
b = -26.8
95% CI = [-32.67, -20.94]
0.5 SD (PTCL = 60)
b = -33.43
95% CI = [-39.99, -26.88]
1 SD (PTCL = 80)
b = -40.06
95% CI = [-48.36, -31.77]
RT
1000
800
600
400
-1.0
-0.5
0.0
0.5
1.0
1.5
WMC

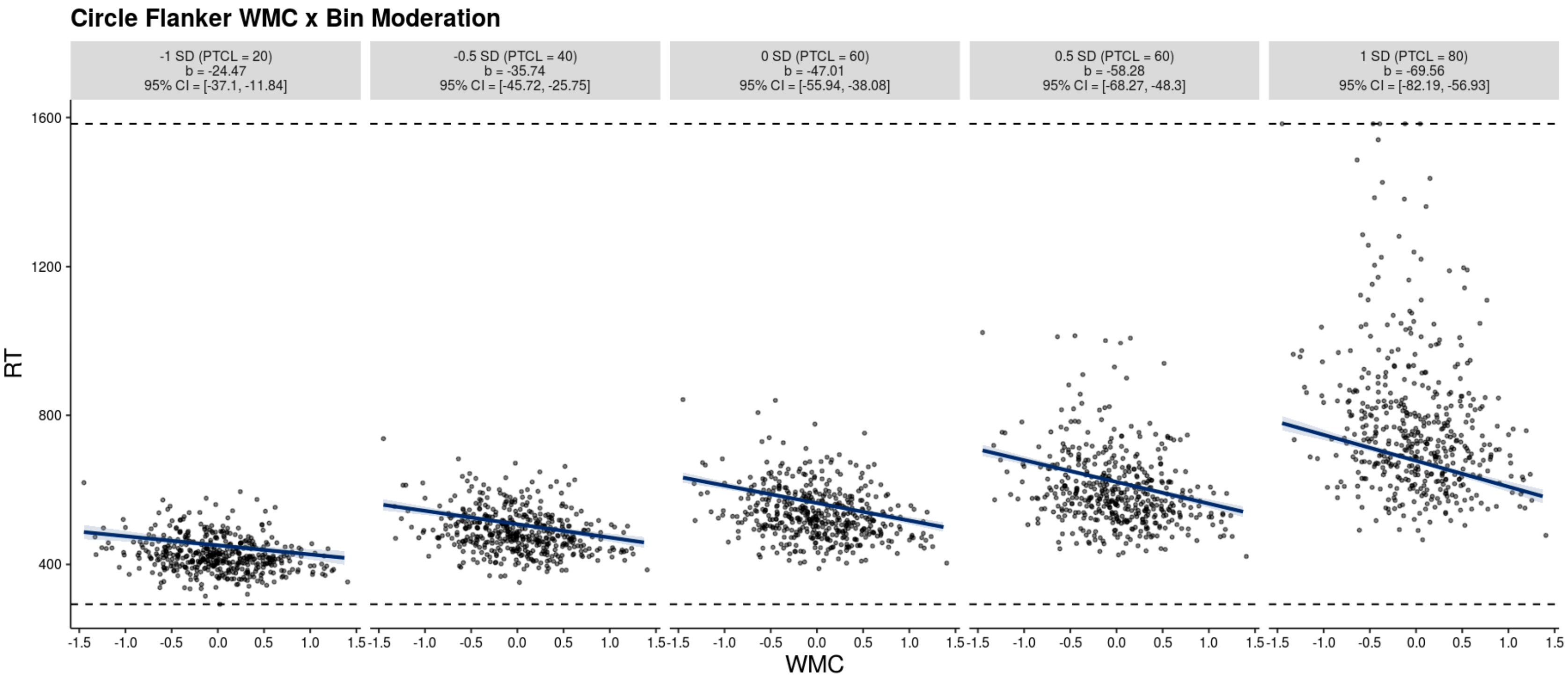
Circle Flanker WMC x Bin Moderation
-1 SD (PTCL = 20)
b = -24.47
95% CI = [-37.1, -11.84]
-0.5 SD (PTCL = 40)
b = -35.74
95% CI = [-45.72, -25.75]
0 SD (PTCL = 60)
b = -47.01
95% CI = [-55.94, -38.08]
0.5 SD (PTCL = 60)
b = -58.28
95% CI = [-68.27, -48.3]
1 SD (PTCL = 80)
b = -69.56
95% CI = [-82.19, -56.93]
RT
1600
1200
800
400
-1.5
-1.0
-0.5
0.0
0.5
1.0
1.5
WMC

Supplemental Figure 2. RT predicted by TUT x Bin for each task.

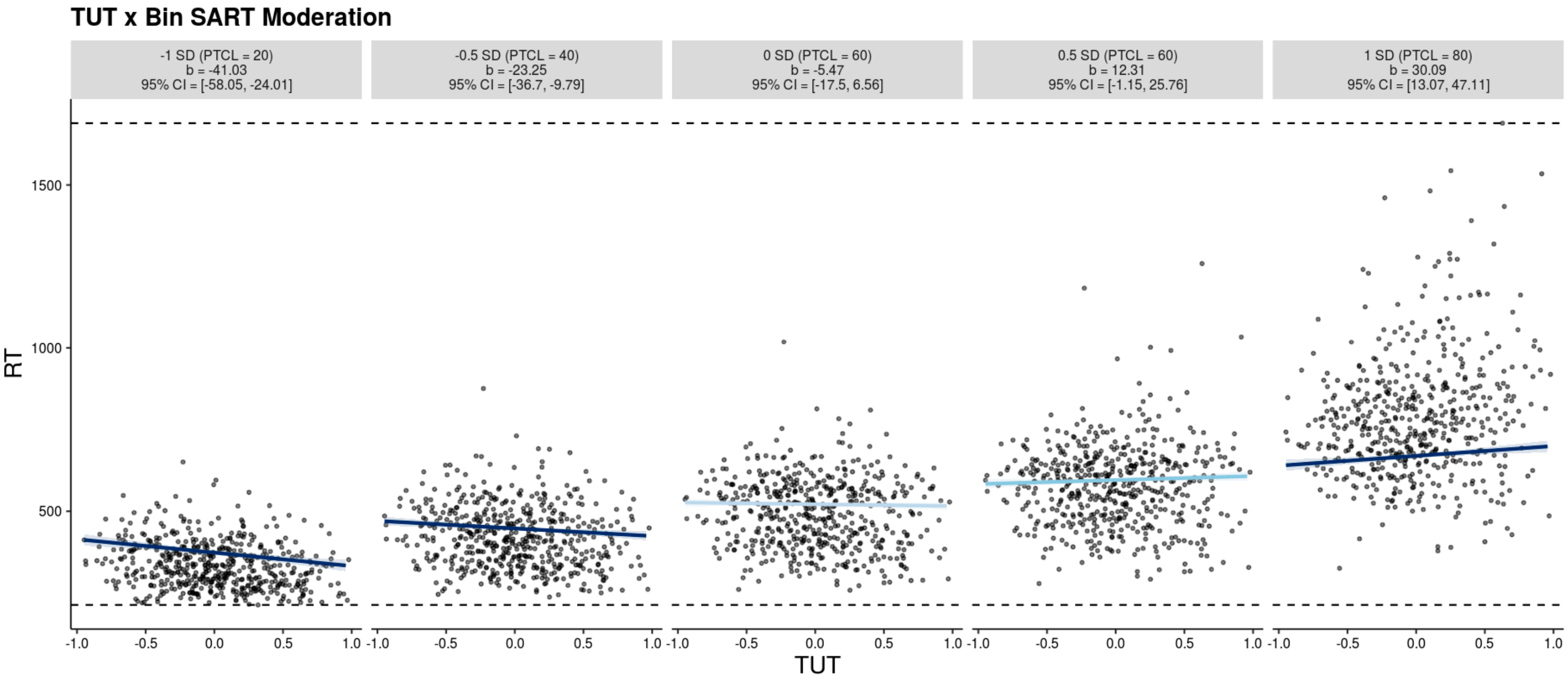

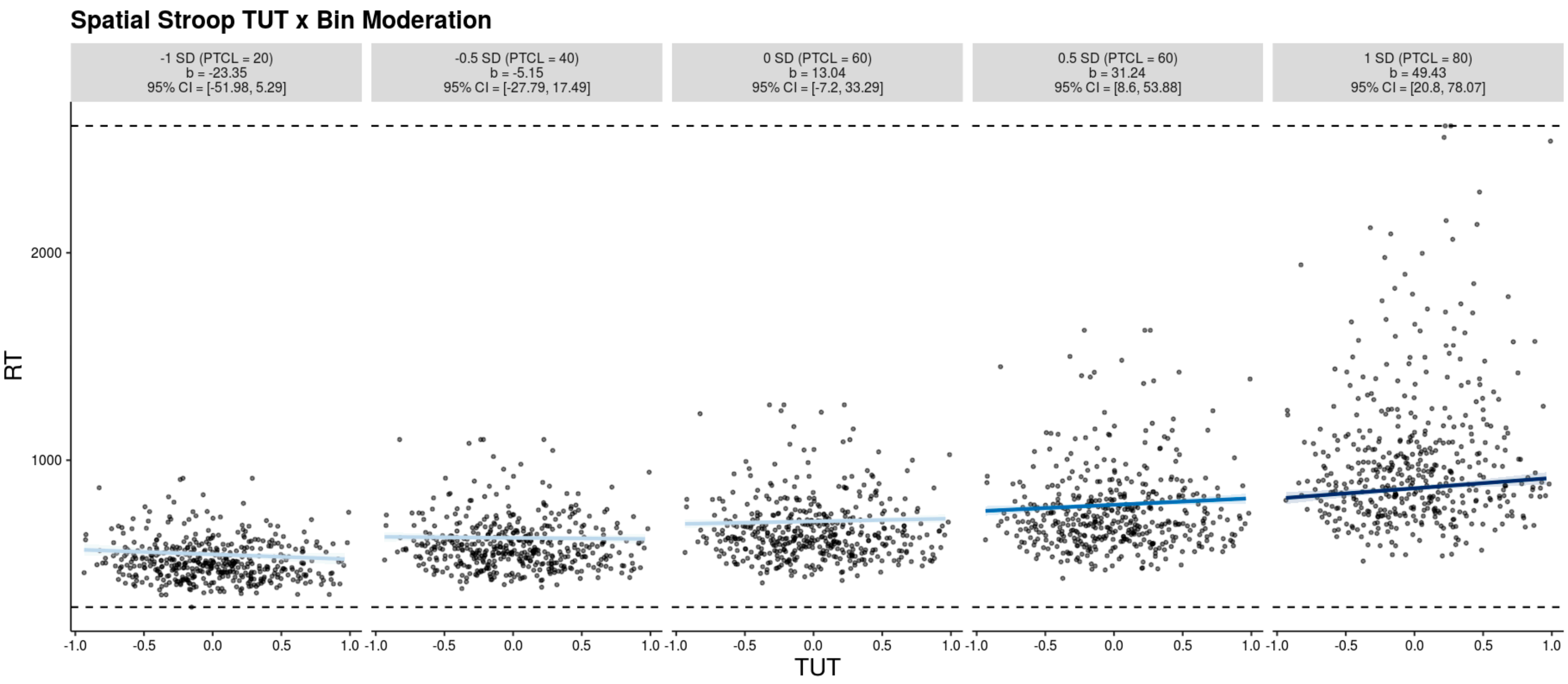

Spatial Stroop TUT x Bin Moderation
-1 SD (PTCL = 20)
b = -23.35
95% CI = [-51.98, 5.29]
-0.5 SD (PTCL = 40)
b = -5.15
95% CI = [-27.79, 17.49]
0 SD (PTCL = 60)
b = 13.04
95% CI = [-7.2, 33.29]
0.5 SD (PTCL = 60)
b = 31.24
95% CI = [8.6, 53.88]
1 SD (PTCL = 80)
b = 49.43
95% CI = [20.8, 78.07]
RT
2000
1000
-1.0
-0.5
0.0
0.5
1.0
TUT

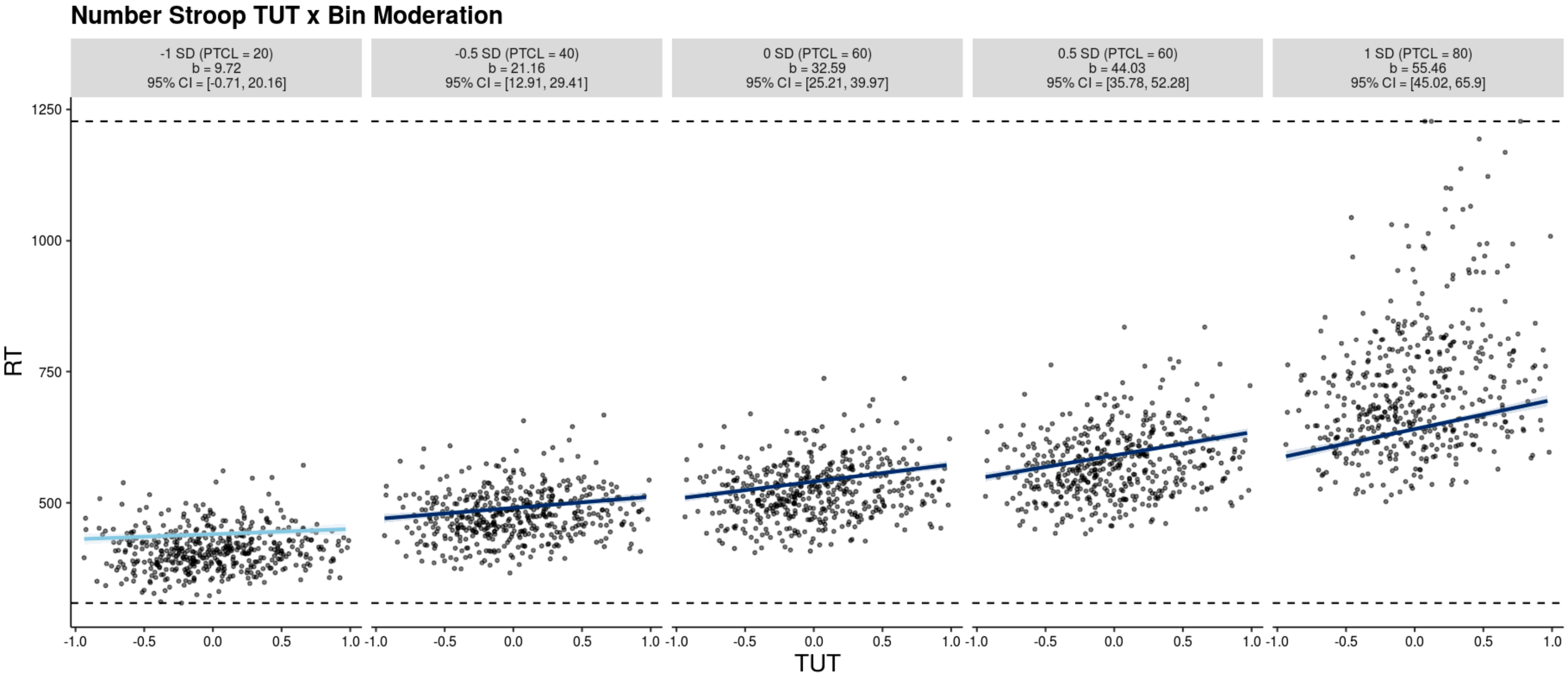
Number Stroop TUT x Bin Moderation
-1 SD (PTCL = 20)
b = 9.72
95% CI = [-0.71, 20.16]
-0.5 SD (PTCL = 40)
b = 21.16
95% CI = [12.91, 29.41]
0 SD (PTCL = 60)
b = 32.59
95% CI = [25.21, 39.97]
0.5 SD (PTCL = 60)
b = 44.03
95% CI = [35.78, 52.28]
1 SD (PTCL = 80)
b = 55.46
95% CI = [45.02, 65.9]
RT
1250
1000
750
500
-1.0
-0.5
0.0
0.5
1.0
TUT

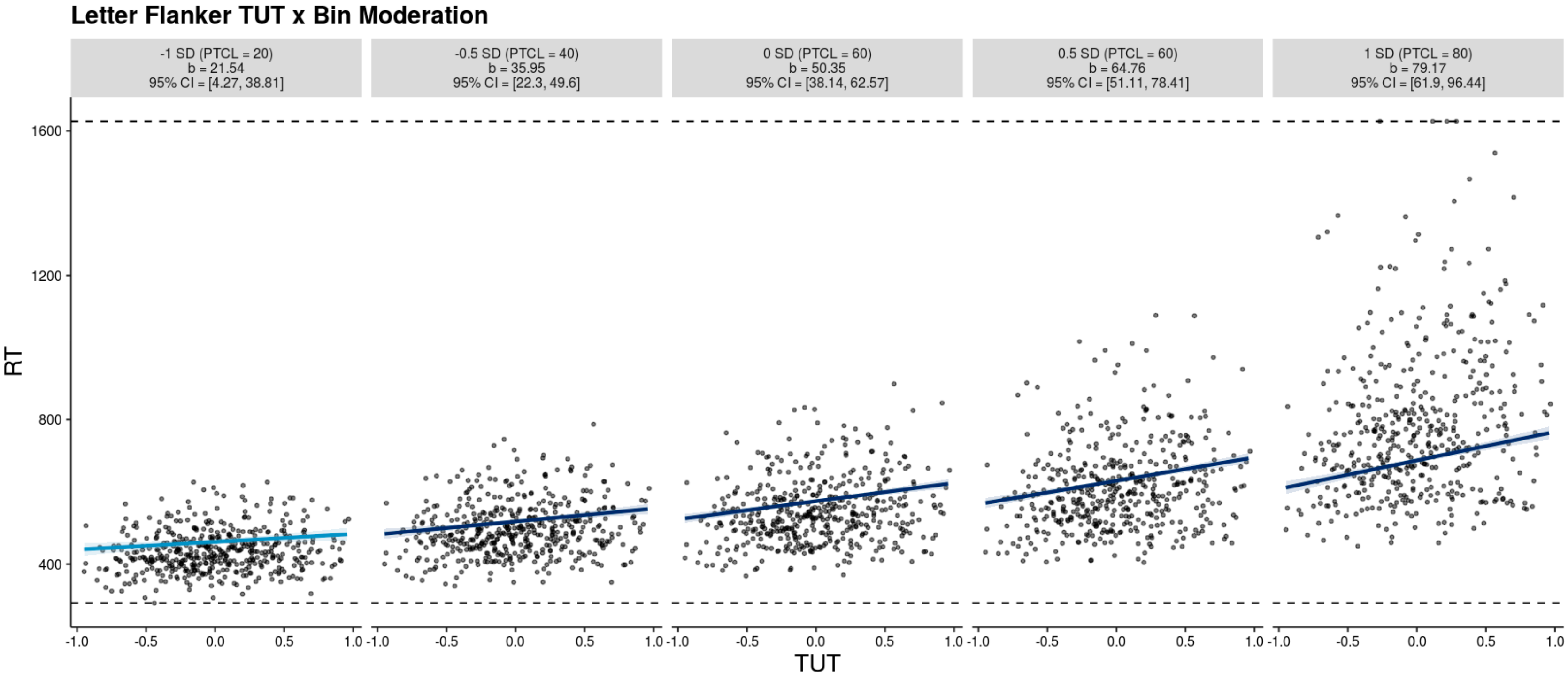
Letter Flanker TUT x Bin Moderation
-1 SD (PTCL = 20)
b = 21.54
95% CI = [4.27, 38.81]
-0.5 SD (PTCL = 40)
b = 35.95
95% CI = [22.3, 49.6]
0 SD (PTCL = 60)
b = 50.35
95% CI = [38.14, 62.57]
0.5 SD (PTCL = 60)
b = 64.76
95% CI = [51.11, 78.41]
1 SD (PTCL = 80)
b = 79.17
95% CI = [61.9, 96.44]
RT
1600
1200
800
400
-1.0
-0.5
0.0
0.5
1.0
TUT

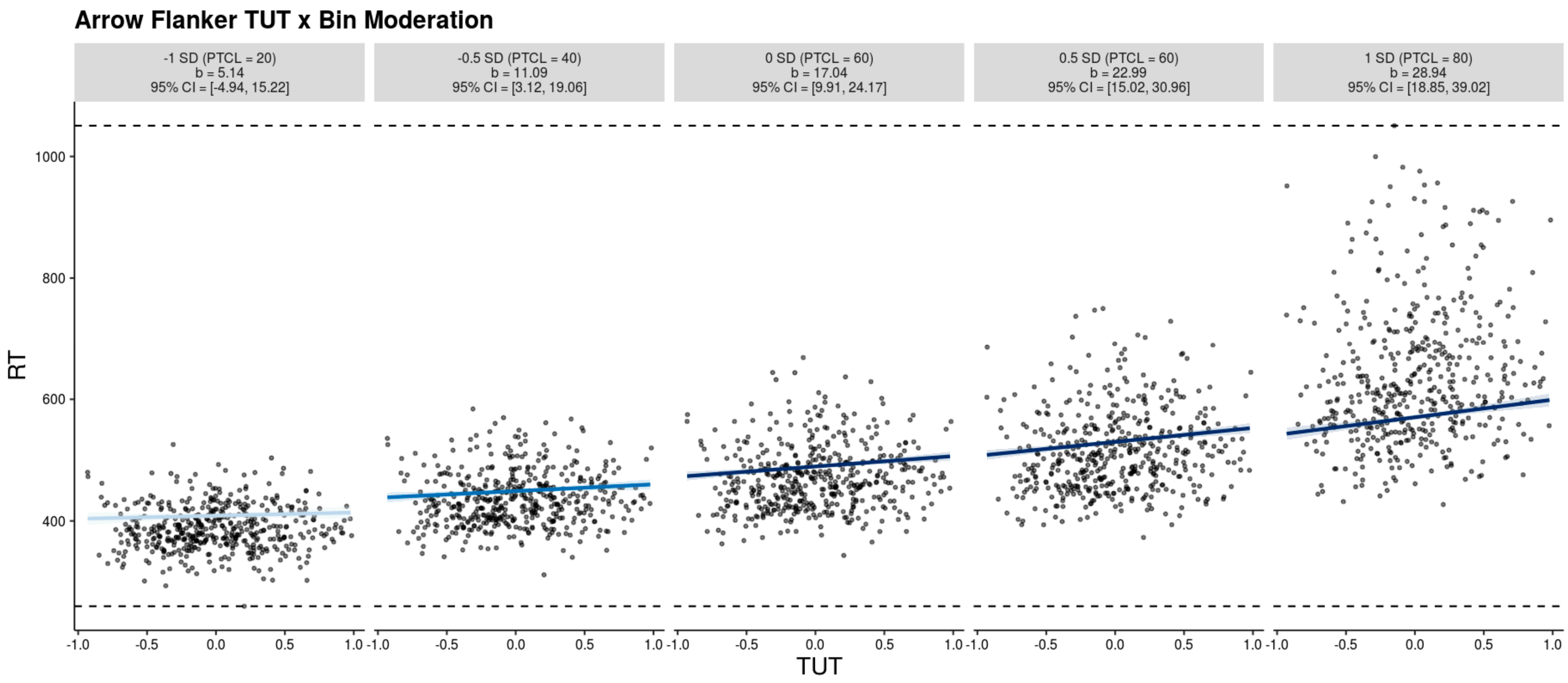
Arrow Flanker TUT x Bin Moderation
-1 SD (PTCL = 20)
b = 5.14
95% CI = [-4.94, 15.22]
-0.5 SD (PTCL = 40)
b = 11.09
95% CI = [3.12, 19.06]
0 SD (PTCL = 60)
b = 17.04
95% CI = [9.91, 24.17]
0.5 SD (PTCL = 60)
b = 22.99
95% CI = [15.02, 30.96]
1 SD (PTCL = 80)
b = 28.94
95% CI = [18.85, 39.02]
RT
1000
800
600
400
-1.0
-0.5
0.0
0.5
1.0
TUT

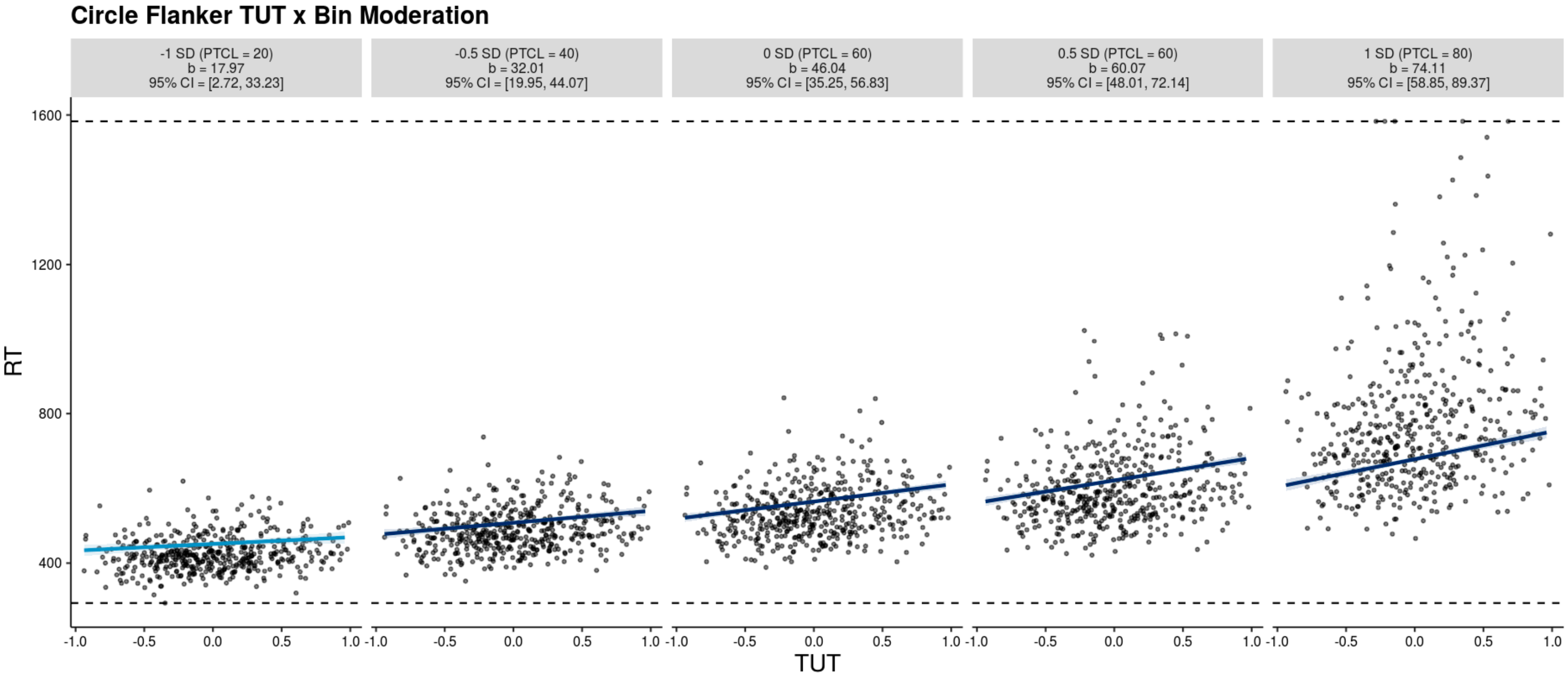
Circle Flanker TUT x Bin Moderation
-1 SD (PTCL = 20)
b = 17.97
95% CI = [2.72, 33.23]
-0.5 SD (PTCL = 40)
b = 32.01
95% CI = [19.95, 44.07]
0 SD (PTCL = 60)
b = 46.04
95% CI = [35.25, 56.83]
0.5 SD (PTCL = 60)
b = 60.07
95% CI = [48.01, 72.14]
1 SD (PTCL = 80)
b = 74.11
95% CI = [58.85, 89.37]
RT
1600
1200
800
400
-1.0
-0.5
0.0
0.5
1.0
TUT
